# Autophagy inhibitor-sensitized artificially activated neutrophils against hepatocellular carcinoma

**DOI:** 10.7150/thno.106404

**Published:** 2025-06-18

**Authors:** Caixia Yang, Huang Yang, Zhengwei Mao, Weilin Wang, Yuan Ding

**Affiliations:** 1 Department of Hepatobiliary and Pancreatic Surgery, the Second Affiliated Hospital, Zhejiang University School of Medicine, Hangzhou, Zhejiang 310009, China.; 2 Key Laboratory of Precision Diagnosis and Treatment for Hepatobiliary and Pancreatic Tumor of Zhejiang Province, Hangzhou, Zhejiang 310009, China.; 3 Research Center of Diagnosis and Treatment Technology for Hepatocellular Carcinoma of Zhejiang Province, Hangzhou, Zhejiang 310009, China.; 4 Center for Medical Research and Innovation in Digestive System Tumors, Ministry of Education, Hangzhou, Zhejiang 310009, China.; 5 Cancer Center, Zhejiang University, Hangzhou, Zhejiang 310058, China.; 6 MOE Key Laboratory of Macromolecular Synthesis and Functionalization, Department of Polymer Science and Engineering, Zhejiang University, Hangzhou, Zhejiang, China.

**Keywords:** Artificial activated neutrophil, Autophagy, Sensitization, Iron, HCC

## Abstract

The use of activated neutrophils has emerged as a promising antineoplastic method in oncology. However, challenges, including a short lifespan, susceptibility to the tumor microenvironment, and protumorigenic risks, limit their clinical application. While artificial neutrophils have several limitations, few tumor-related studies have been conducted with constraining factors, including specific targeting inefficiency, immunogenicity and manufacturing challenges. Neutrophil elastase (ELANE), a key antitumor effector in activated neutrophils, is functionally mimicked by porcine pancreatic elastase (PPE), which exhibits selective cancer cytotoxicity. However, PPE triggers protective autophagy in hepatocellular carcinoma (HCC), limiting its therapeutic effectiveness.

**Methods**: To overcome this resistance, we sensitized PPE by the autophagy inhibitor 3-methyladenine (3MA), which is codelivered via tumor-targeting liposomes. This system protects drugs and improves therapeutic efficacy both *in vitro* and *in vivo*.

**Results**: 3MA enhanced iron-related ROS-mediated cell destruction induced by PPE while suppressing prosurvival autophagy. The autophagy inhibitor-sensitized artificially activated neutrophils (asAN-P/3) showed precise tumor targeting, excellent therapeutic efficacy, prolonged survival and favorable biocompatibility.

**Conclusions**: We established a precise neutrophil-related tumor therapeutic method (asAN-P/3) and elucidated the mechanistic insights into PPE-mediated therapeutic limitations in HCC. Our study provides a substantial framework for the development of neutrophil-derived antitumor therapeutic strategies in oncology.

## Introduction

Hepatocellular carcinoma (HCC), the sixth most common malignancy and the third leading cause of cancer mortality globally. The associated risk factors include virus infections 1, nonalcoholic fatty liver disease, alcohol-associated cirrhosis and iron overload 2. For early-stage HCC, a significant proportion of patients (approximately 30-50%) experience tumor recurrence within 3 years 3. For advanced stages, suboptimal drug efficacy and adverse effects limit treatment efficacy 4. Therefore, further research is imperative to develop novel and effective treatment modalities for HCC.

Activated neutrophils have emerged as promising antineoplastic agents because of their specific antitumor ability 5. However, neutrophil-related therapy has a high demand for cells from patients themselves, which may cause side effects 6. Moreover, their clinical translation has been hindered by protumorigenic plasticity 5, short lifespan, logistical challenges in cell sourcing and resistance to genome editing 7,8.

Research related to artificial neutrophils has focused primarily on inflammatory treatments, with less attention given to their application in oncology 9. Research in oncology has focused primarily on neutrophil membranes, whereas immune cell membranes are derived mainly from immortalized cells, which may lead to undesirable biological effects. Furthermore, the targeting ability of neutrophil membranes depends on the level of inflammatory chemotaxis signaling in the tumor 6. Additionally, membrane immunogenicity, the immune response to denatured proteins produced during preparation, the integrity of membrane proteins, and the costs of production all constrain their application 10.

Therefore, there is a need for more efficient, stable, specific, safe and cost-controllable methods for artificial neutrophil-related antitumor therapy.

Recent studies have demonstrated that activated neutrophils exert antitumor effects primarily through neutrophil elastase (ELANE). It is internalized into tumor cells and selectively induces apoptosis 11,12. Porcine pancreatic elastase (PPE), a promising cost-effective alternative to ELANE 11,13, can be constrained by natural protease inhibitors in the blood 14,15, extracellular connective tissue 16, and the nontargeted distribution and availability of cellular receptors 17-19.

Therefore, we attempted to utilize biocompatible liposomes to create artificially activated neutrophils to protect them 20. Considering the limited targeting ability of liposomes, we integrated the tumor-homing cyclic RGD (cRGD) peptide as a critical component to guide the chemotaxis of artificially activated neutrophils to tumor tissues. It can specifically bind to αvβ3 integrins that are overexpressed in tumor cells 21, increasing the stability of the targeting ability of artificially activated neutrophils. Moreover, it is easy to synthesize and has broader applicability, facilitating its clinical translation.

Critically, we identified a limitation in PPE-mediated therapy for HCC, attributed to the activation of autophagy. Autophagy, a prosurvival mechanism in cancer cells, is associated with resistance to cancer therapies, protecting tumors from death 22.

Therefore, enhancing the effectiveness of PPE by inhibiting autophagy is worthwhile. 3-Methyladenine (3MA) is a classical early-stage autophagy inhibitor that targets phospholipase-like III enzymes (PI3K). It has shown broad applicability in tumor treatments with a favorable safety profile 23,24. Subsequently, we incorporated 3-MA to sensitize artificially activated neutrophils. Consequently, we developed autophagy inhibitor-sensitized artificially activated neutrophils (asAN-P/3) with excellent targeting capability for HCC therapy.

**Scheme 1:**
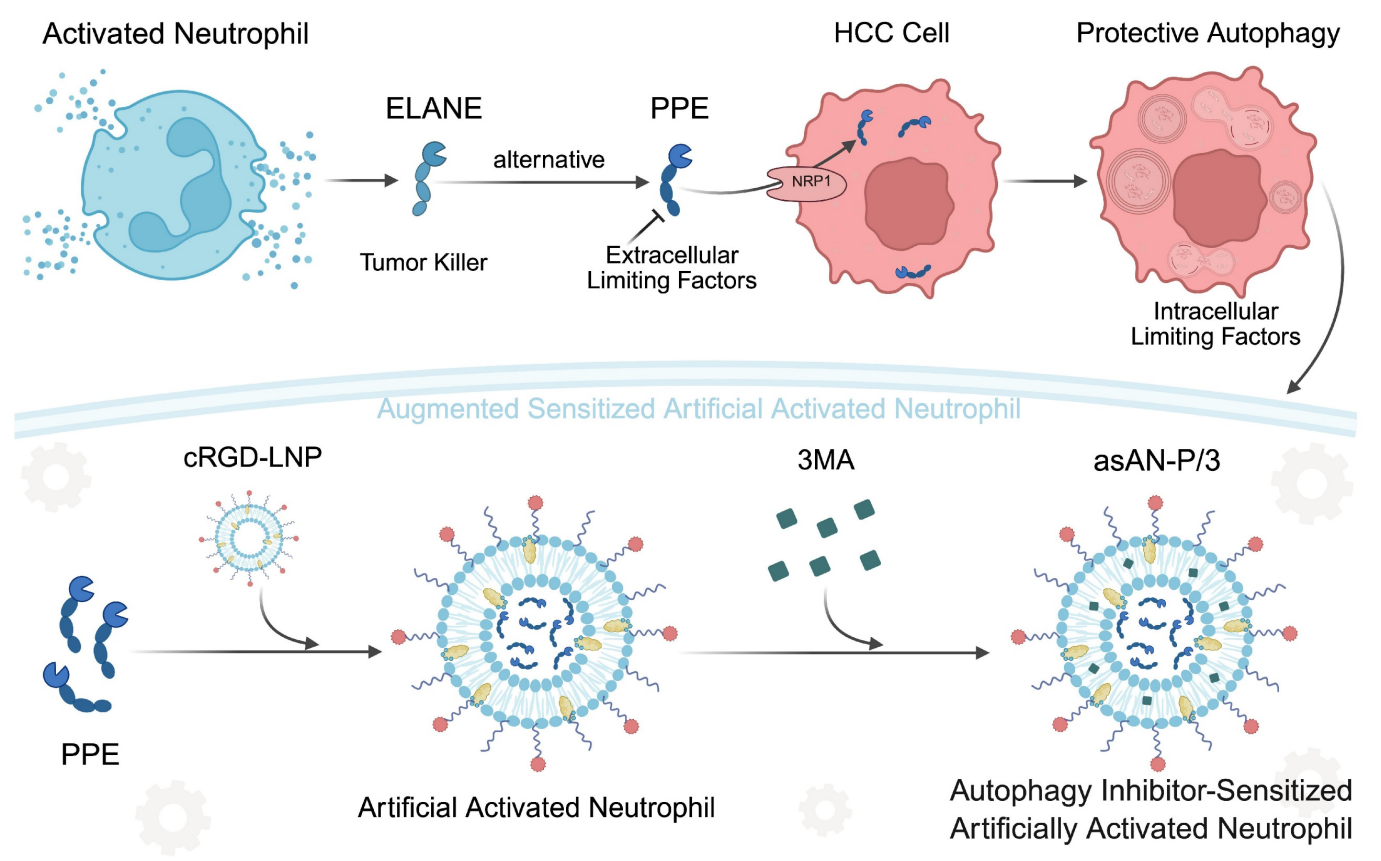
** Schematic illustration of the autophagy inhibitor-sensitized artificially activated neutrophils (asAN-P/3) designed for HCC therapy.** ELANE serves as a tumor killer in activated neutrophils, with selective cytotoxicity in cancer. PPE is a promising alternative to ELANE. However, protective autophagy, receptor availability (intracellular), blood, extracellular connective tissue and nontargeted (extracellular) factors limit PPE-mediated therapeutic effectiveness against HCC. Based on the concept of artificially activated neutrophils, which are characterized by cRGD-modified liposomes loaded with PPE, 3-MA was subsequently incorporated to sensitize artificially activated neutrophils. Consequently, we developed asAN-P/3 with stable targeting capability for HCC therapy. cRGD-LNP: cRGD-modified liposomes; asAN-P/3: autophagy inhibitor-sensitized artificially activated neutrophils.

In this work, we discovered the limited therapeutic efficacy of the key activated neutrophil-killing effector mimicker (PPE) in HCC, which is characterized by activated protective autophagy. Therefore, 3MA was employed to increase the therapeutic efficacy of PPE. The combination therapy disrupted iron-related ROS homeostasis and autophagy-mediated survival, synergistically enhancing apoptosis. Subsequently, we developed autophagy inhibitor-sensitized artificially activated neutrophils (asAN-P/3) for HCC therapy **(Scheme 1)**. It exhibited robust antitumor efficacy and prolonged survival *in vitro* and *in vivo*, with precise tumor targeting and biocompatibility. As a consequence, this work contributes valuable insights for the advancement of emerging neutrophil-related therapeutics in oncology.

## Experimental Methods

### Materials

All reagents and solvents were obtained commercially and utilized without subsequent purification. Phosphate buffer solution (PBS), DMEM, fetal bovine serum and penicillin‒streptomycin (100X) were purchased from Gibco (USA). 3-Methyladenine (3MA), N-acetylcysteine (NAC), the Cell Counting Kit-8 (CCK8) and tetramethylrhodamine ethyl ester (TMRE) were purchased from MCE (China). Porcine pancreatic elastase (PPE) was purchased from Worthington (USA). Chloroquine (CQ), deferoxamine (DFO) and N-methoxysuccinyl-Ala-Ala-Pro-Val p-nitroanilide (elastase substrate) were purchased from Sigma‒Aldrich (USA). Cholesterol was purchased from Aladdin (China). DSPE-PEG2000 was purchased from Ruixibio (China). Lecithin was purchased from Yuanye Bio Technology (China). Cyclo(RGD-Gly-C16) (cRGD) was purchased from Nanjing Yuanpeptide Biotechnology Co., Ltd. (Nanjing, Jiangsu). Anti-Nrf2 (Cat# ab62352), anti-CD71 (Cat# ab214039), anti-ferritin (Cat# ab75973), anti-IRS1 (Cat# ab40777), anti-LC3B (Cat# ab192890), anti-Ki67 (Cat# ab15580) and anti-DMT1 (Cat# ab55735) antibodies were purchased from Abcam (UK). Anti-ATG7 (Cat# 8558S), anti-ATG5 (Cat# 12994S), anti-cleaved caspase 3 (Asp175) (Cat# 9661T), anti-p62 (Cat# 88588S), anti-HIF1α (Cat# 14179s) and anti-s-XBP1 (Cat# 40435s) antibodies were purchased from Cell Signaling Technology (USA). Anti-β-actin (Cat# 20536-1-AP) and anti-GAPDH (Cat# 60004-1-Ig) antibodies were purchased from Proteintech (USA). Anti-tubulin (Cat# A12289) was purchased from ABclonal (China). The LDH Cytotoxicity Assay Kit, BeyoClick™ EdU Cell Proliferation Kit with Alexa Fluor 488, Ad-mCherry-GFP-LC3B and One Step TUNEL Apoptosis Assay Kit were purchased from Beyotime (China). A calcein-AM/PI live/dead cell staining kit was purchased from Solarbio (China). A cell cycle staining kit was purchased from MULTI SCIENCES (China). CM-H2DCFDA (DCFH), Hoechst 33342 and DAPI were purchased from Thermo Fisher Scientific (USA). FerroOrange, the Annexin V-FITC Apoptosis Detection Kit, the SOD Assay Kit and the GSSG/GSH Quantification Kit II were purchased from Dojindo (Japan).

### Cells and mice

The human hepatocellular carcinoma cell line Hep-3B was obtained from Percell (China). The cells were maintained in DMEM supplemented with 10% fetal bovine serum and 1% penicillin‒streptomycin solution at 37°C in an atmosphere containing 5% CO2. The animal experimental protocol received approval from the Institutional Animal Care and Use Committee of Zhejiang Center of Laboratory Animals (ZJCLA) (ZJCLA-IACUC-20010242). Wild-type male BALB/c mice (GemPharmatech, China) aged 5 weeks were used for all *in vivo* experiments. The mice were housed under standard conditions, with an ambient room temperature of 22 ± 2°C, humidity ranging from 40% to 70%, and a 12-hour light/dark cycle.

### Gene databases

Autophagy-related gene expression profile datasets were downloaded from the Human Autophagy Database (HADb) 27. Iron metabolism-related gene expression profile datasets were downloaded from the article (doi: 10.3389/fnagi.2022.949083) reported by Xuefeng Gu and Donglin Lai, *et al.* 51.

### *In vitro* analysis of the therapeutic efficacy and functional mechanisms of drugs

#### Cell viability assay

4000 per well Hep-3B cells were seeded into 96-well plates and cultured for 12 h. Then, the Hep-3B cells were subjected to the indicated drug formulations for 24 h (1. different concentrations of PPE; 2. 40 μg/mL PPE combined with different concentrations of 3MA) under serum-free conditions. To assess cell viability, CCK8 analysis was performed by microplate reader using the CCK8 reagent following the protocol provided by the manufacturer.

#### Cell proliferation assay

1.5×10^5^ per well Hep-3B cells were seeded into confocal imaging culture dishes and cultured for 12 h. Then, the Hep-3B cells were subjected to the indicated drug formulations for 22 h (PBS, PPE: 40 μg/mL) under serum-free conditions. Then, an equal volume of EdU working solution preheated at 37°C was added, and the mixture was incubated for 2 h. To assess cell proliferation, fluorescence imaging analysis was performed by confocal laser scanning microscope using the BeyoClick™ EdU Cell Proliferation Kit with Alexa Fluor 488 and DAPI, following the protocol provided by the manufacturer.

#### Cell cycle assay

4.0×10^5^ per well Hep-3B cells were seeded into 60 mm cell culture dishes and cultured for 12 h. Then, the Hep-3B cells were subjected to the indicated drug formulations for 24 h (PBS, PPE: 40 μg/mL) under serum-free conditions. Then, the cells were collected and fixed with precooled 70% ethanol for 12-24 h. To assess the cell cycle distribution, flow cytometry analysis was performed by flow cytometry using the Cell Cycle Staining Kit following the protocol provided by the manufacturer.

#### Lactate dehydrogenase (LDH) release assay

4000 per well Hep-3B cells were seeded into 96-well plates and cultured for 12 h. Then, the Hep-3B cells were subjected to the indicated drug formulations for 24 h (different concentrations of PPE) under serum-free conditions. To assess cellular toxicity, extracellular LDH concentration analysis was performed by microplate reader using the LDH Cytotoxicity Assay Kit following the protocol provided by the manufacturer.

#### Cell death or viability assay

1.5×10^5^ per well Hep-3B cells were seeded into confocal imaging culture dishes and cultured for 12 h. Then, the Hep-3B cells were subjected to the indicated drug formulations for 24 h (1. different concentrations of PPE; 2. PBS, PPE: 40 μg/mL, 3MA: 5 mM, NAC: 5 mM) under serum-free conditions. To assess cell toxicity, fluorescence imaging analysis was performed by confocal laser scanning microscopy via a calcein-AM/PI live/dead cell staining kit following the protocol provided by the manufacturer.

#### Cell death and apoptosis assay

2.0×10^5^ per well Hep-3B cells were seeded into 6-well plates and cultured for 12 h. Then, the Hep-3B cells were subjected to the indicated drug formulations for 48 h (1. PBS, PPE: 40 μg/mL; 2. PBS, PPE: 40 μg/mL, 3MA: 5 mM) under serum-free conditions. To assess cell death and apoptosis, flow cytometry analysis was performed by flow cytometry using the Annexin V-FITC Apoptosis Detection Kit following the protocol provided by the manufacturer.

#### Immunoblotting

2.0×10^5^ per well Hep-3B cells were seeded into 6-well plates and cultured for 12 h. Then, the Hep-3B cells were subjected to the indicated drug formulations (autophagy: PBS, PPE: 40 μg/mL, 3MA: 0.5 mM, CQ: 25 μM; apoptosis: PBS, PPE: 40 μg/mL, 3MA: 5 mM; iron metabolism and IRS1: PBS, PPE: 40 μg/mL, 3MA: 5 mM) for 16-24 h (apoptosis: cleaved caspase 3 24 h; autophagy, iron metabolism and IRS1: 16 h) under serum-free conditions. Then, the cells were lysed. Protein lysates were separated by SDS‒PAGE and subsequently transferred to 0.22-0.45 μm NC membranes. After that, the samples were subjected to standard immunoblotting procedures as previously described.

#### Autophagy assay

1.0×10^5^ per well Hep-3B cells were seeded into confocal imaging culture dishes. When the cell density reached approximately 50%, Ad-mCherry-GFP-LC3B was added, and the cells were infected for 24 h. Then, the cells were removed, and the infected Hep-3B cells were subjected to the indicated drug formulations for 16 h (PBS, PPE: 40 μg/mL, 3MA: 0.5 mM, CQ: 25 μM) under serum-free conditions. To assess autophagy activation, fluorescence imaging analysis was performed by confocal laser scanning microscopy after staining with Hoechst 33342 following the protocol provided by the manufacturer.

#### Cell apoptosis assay by mitochondrial membrane potential analysis

2.0×10^5^ per well Hep-3B cells were seeded into 6-well plates and cultured for 12 h. Then, the Hep-3B cells were subjected to the indicated drug formulations for 24 h (PBS, PPE: 40 μg/mL, 3MA: 5 mM) under serum-free conditions. To assess cell apoptosis, flow cytometry analysis was performed by flow cytometry using TMRE following the protocol provided by the manufacturer.

#### ROS quantitative assay

2.0×10^5^ per well Hep-3B cells were seeded into 6-well plates and cultured for 12 h. Then, the Hep-3B cells were subjected to the indicated drug formulations for 16 h (PBS, PPE: 40 μg/mL, 3MA: 5 mM) under serum-free conditions. To assess the ROS level, flow cytometry analysis was performed by flow cytometry using CM-H2DCFDA (DCFH) following the protocol provided by the manufacturer.

#### SOD inhibition rate assay

4×10^5^ per well Hep-3B cells were seeded into 60 mm cell culture dishes and cultured for 12 h. Then, the Hep-3B cells were subjected to the indicated drug formulations for 16 h (PBS, PPE: 40 μg/mL, 3MA: 5 mM) under serum-free conditions. To assess SOD activity, the intracellular SOD inhibition rate was analyzed by microplate reader using SOD Assay Kit following the protocol provided by the manufacturer.

#### GSH concentration assay

4×10^5^ per well Hep-3B cells were seeded into 60 mm cell culture dishes and cultured for 12 h. Then, the Hep-3B cells were subjected to the indicated drug formulations for 16 h (PBS, PPE: 40 μg/mL, 3MA: 5 mM) under serum-free conditions. To assess GSH activity, intracellular GSH concentration analysis was performed by microplate reader using GSSG/GSH Quantification Kit II following the protocol provided by the manufacturer.

#### Intracellular ferrous ion assay

1.5×10^5^ per well Hep-3B cells were seeded into confocal imaging culture dishes and cultured for 12 h. Then, the Hep-3B cells were subjected to the indicated drug formulations for 16 h (PBS, PPE: 40 μg/mL, 3MA: 5 mM) under serum-free conditions. To assess the ferrous ion concentration, fluorescence imaging analysis was performed by confocal laser scanning microscopy using FerroOrange, following the protocol provided by the manufacturer.

#### Intracellular ROS assay

1.5×10^5^ per well Hep-3B cells were seeded into confocal imaging culture dishes and cultured for 12 h. Then, the Hep-3B cells were subjected to the indicated drug formulations for 16 h (PBS, PPE: 40 μg/mL, 3MA: 5 mM, DFO: 100 μM) under serum-free conditions. To assess the ROS concentration, fluorescence imaging analysis was performed by confocal laser scanning microscopy using CM-H2DCFDA (DCFH) following the protocol provided by the manufacturer.

### Bioinformatics analysis

#### Acquisition of transcriptome data from cells

5×10^6^ Hep-3B cells subjected to the indicated drug formulations for 16 h (PBS, PPE: 40 μg/mL, under serum-free conditions) were collected. To acquire the transcriptome data, 1 mL of TRIzol was used to lyse the cells. The lysed samples were subsequently transported to Cosmos Wisdom for transcriptome sequencing.

#### Differentially expressed gene analysis

The online analysis tool GEO2R was used to identify the differentially expressed genes (DEGs). Genes with the specific cutoff criteria of ∣log2(fold change)∣ > 0 and adjusted *p* < 0.05 were considered DEGs, and a volcano plot was generated with the R package. Genes whose log2(fold change) > 0 were considered upregulated genes, whereas those whose log2(fold change) < 0 were considered downregulated genes.

The online database Sangerbox (http://vip.sangerbox.com/home.html) was used to assess the functions of differentially expressed genes by conducting gene ontology (GO) analysis and Kyoto Encyclopedia of Genes and Genomes (KEGG) pathway enrichment analysis. GO analysis revealed three fundamental aspects: biological process (BP), cellular component (CC) and molecular function (MF). The significance threshold was set at a *Q* value < 0.05, and an enriched gene count ≥ 5 was chosen as the criterion for statistical significance.

The DEGs in the PPE group and the datasets from the HADb database were merged to identify the overlapping DEGs. A visual hierarchical cluster analysis Venn diagram and heatmap were generated with the R package. To evaluate the protein‒protein interactions between the identified differentially expressed genes, the online database STRING (https://string-db.org/) was used to perform protein‒protein interaction (PPI) network analysis.

A list of genes identified through iron metabolism-related datasets^51^ was selected. Gene set enrichment analysis (GSEA) was conducted with the R package, and adjusted *p* < 0.05 was set as the criterion for statistical significance. The gene set variation analysis (GSVA) algorithm was subsequently used to score the gene sets with the R package to evaluate the prospective alterations in biological functionality.

### Preparation and characterization of nanoformulations

#### Preparation of nanoformulations

Initially, 4 mg of C16-cRGD was dissolved in 4 mL of a trifluoroacetic acid and acetonitrile mixture (10:90, v/v) and subsequently transferred to a 50 mL round-bottom flask for rotary evaporation to produce a film. A second film was subsequently formed by 12 mg of lecithin, 4 mg of cholesterol, and 4 mg of DSPE-PEG in 2 mL of chloroform in the aforementioned flask. Similarly, a third film was created by dissolving 3 mg of 3MA in 3 mL of ethanol. Furthermore, 1 mg of PPE was dissolved in 1 mL of PBS buffer and introduced into the flask following ultrasonic vibration. The resulting asAN-P/3 was purified using a dialysis bag (MWCO 300 kDa).

#### Determination of the 3MA ratio in asAN-P/3

4000 per well Hep-3B cells were seeded into 96-well plates and cultured for 12 h. Hep-3B cells were subsequently subjected to the indicated drug formulations for 24 h (asAN-P/3 with different ratios of 3MA/PPE, in which PPE was 25 μg/mL) in the presence of serum. To assess cell viability, CCK8 analysis was performed by microplate reader using the CCK8 reagent following the protocol provided by the manufacturer.

#### Determination of the cRGD ratio in asAN-P/3

1.5×10^5^ per well Hep-3B cells were seeded into confocal imaging culture dishes and cultured for 12 h. Then, the Hep-3B cells were subjected to the indicated drug formulations for 1 h (PBS and treatments with different ratios of cRGD/PPE in asAN-P/3, in which PPE was 20 μg/mL) in the presence of serum. To assess cellular uptake ability, fluorescence imaging analysis was performed by confocal laser scanning microscopy after staining with Hoechst 33342 following the protocol provided by the manufacturer.

#### Assessment of cRGD density in liposomes

1 mL of C16-cRGD solution (2 mg/mL, dissolved in a trifluoroacetic acid and acetonitrile mixture) and 10 μL of Rhodamine B NHS ester (1 mg/mL, dissolved in water) were mixed and cocultured at 37°C for 2 hours. The reaction product (C16-cRGD^RB^) was purified by dialysis. The standard curve was then detected by ultraviolet‒visible light spectroscopy. 1.5 mg/mL liposomes prepared by C16-cRGD^RB^ were diluted 5-fold by mixed solution of trifluoroacetic acid and acetonitrile. The cRGD density in liposomes was assessed by the ultraviolet‒visible light spectrum of the liposome solution.

#### Measurement of the zeta potential, size and stability of the nanoformulations

The size and zeta potential of asAN-P/3 were determined using Zetasizer Nano ZS90, and the morphology of the nanoparticles was characterized via transmission electron microscopy. The stability of asAN-P/3 was assessed by monitoring size changes at various time intervals.

#### Stability detection of asAN-P/3 in plasma

0.1 mL of asAN-P/3 was added to 0.9 mL of plasma and cocultured for different durations at 37°C. The abovementioned mixture was subsequently dialyzed in a dialysis bag for 6 h to eliminate free proteins and some small drugs. Then, the hydrated dimeters of purified asAN-P/3 on different days were detected by Nanoparticle Size and Zeta Potential Analyzer.

#### Measurement of drug loading and encapsulation efficiency

The drug loading and encapsulation efficiency were calculated by analyzing the content of 3MA and PPE in the supernatant of the asAN-P/3 solution following centrifugation (12000 rpm, 30 min, 4°C). The concentration of 3MA was determined using ultraviolet‒visible spectroscopy, whereas the concentration of PPE was quantified by BCA detection kits.

#### Measurement of drug release

To evaluate the drug release profile, 2 mL of asAN-P/3 was enclosed in a dialysis bag and immersed in 20 mL of PBS buffer containing Tween 80 (0.1%, w/v) at 37°C under oscillation conditions. At designated time points, 1 mL of buffer was sampled, and fresh buffer was replenished. The drug release kinetics were determined by analyzing the contents of 3MA and PPE in the collected PBS buffer.

#### Measurement of enzyme activity

A total of 266 μg/mL free PPE and released PPE from asAN-P/3 reacted with the elastase substrate N-methoxysuccinyl-Ala-Ala-Pro-Val p-nitroanilide, respectively, following the protocol provided by the manufacturer. To assess enzyme activity, the absorbance at 410 nm was measured with a microplate reader.

### *In vitro* analysis of tumor cell targeting ability and therapeutic efficacy of the nanoformulations

#### Cellular uptake ability assay

1.5×10^5^ per well Hep-3B cells were seeded into confocal imaging culture dishes and cultured for 12 h. Then, the Hep-3B cells were subjected to the indicated drug formulations for 3 h (PBS, L-P^Dil^ or cL-P^Dil^; the concentration of the PPE in the nanoformulations was 7 μg/mL) in the presence of serum. To assess cellular uptake ability, fluorescence imaging analysis was performed by confocal laser scanning microscopy after staining with Hoechst 33342 following the protocol provided by the manufacturer.

#### Cell viability assay

4000 per well Hep-3B cells were seeded into 96-well plates and cultured for 12 h. Then, the Hep-3B cells were subjected to the indicated drug formulations for 24 h (PBS, free PPE, free 3MA, free PPE+3MA, cL-P, cL-3 and asAN-P/3, the concentration of the nanoformulations depended on PPE (2.50 μg/mL, 5.00 μg/mL, 6.25 μg/mL, 10.00 μg/mL, 12.50 μg/mL, 25.00 μg/mL, 40.00 μg/mL, 50.00 μg/mL), and 3MA was 3.67 times that of PPE) in the presence of serum. To assess cell viability, CCK8 analysis was performed by microplate reader using the CCK8 reagent following the protocol provided by the manufacturer.

#### Immunoblotting

2.0×10^5^ per well Hep-3B cells were seeded into 6-well plates and cultured for 12 h. Then, the Hep-3B cells were subjected to the indicated drug formulations for 16 h (PBS, cL-P, cL-3 or asAN-P/3, the concentration of the PPE in the nanoformulations was 15 μg/mL, and 3MA was 3.67 times that of PPE) in the presence of serum. Then, the cells were lysed. Protein lysates were separated by SDS‒PAGE and transferred to 0.22-0.45 μm NC membranes. After that, the samples were subjected to standard immunoblotting procedures as previously described.

#### Cell death and apoptosis assay

2.0×10^5^ per well Hep-3B cells were seeded into 6-well plates and cultured for 12 h. Then, the Hep-3B cells were subjected to the indicated drug formulations for 24 h (PBS, free PPE+3MA, cL-P, cL-3 and asAN-P/3, the concentration of the PPE in the nanoformulations was 0.01 μg/mL, and 3MA was 3.67 times that of PPE) in the presence of serum. To assess cell death and apoptosis, flow cytometry analysis was performed by flow cytometry using the Annexin V-FITC Apoptosis Detection Kit following the protocol provided by the manufacturer.

### *In vivo* analysis of the tumor targeting and therapeutic efficacy of the nanoformulations

#### *In vivo* tumor targeting and biodistribution assay

Hep-3B tumor-bearing BALB/c mice were generated through the injection of 2 ×10^6^ Hep-3B cells subcutaneously on the right side (*n* = 3). Once the tumor volume reached approximately 80-100 mm^3^, the mice were intravenously administered PPE^ICG^, L-P^ICG^ or cL-P^ICG^ (at a dosage of 30.25 μg for PPE). The fluorescence images of the mice were captured at various time points using an *in vivo* fluorescence imaging system. Finally, the tumor tissue, heart, liver, spleen, lungs and kidneys of the mice were obtained. The fluorescence images of the tissues were captured using an *in vivo* fluorescence imaging system.

#### *In vivo* tumor inhibition

Hep-3B tumor-bearing BALB/c mice were generated through the injection of 2 ×10^6^ Hep-3B cells subcutaneously on the right side. Once the tumor volume reached approximately 100 mm^3^, the Hep-3B tumor-bearing mice were randomly divided into five groups (*n* = 6). On day 0, the mice were intravenously administered PBS, free PPE, free PPE+3MA, cL-P, cL-3 or asAN-P/3. These treatments were repeated every two days for a total of five administrations, with a dosage of 60.5 μg for PPE and 222 μg for 3MA. The tumor volume was calculated using the following standard formula: V = (tumor length) × (tumor width)^2^ /2. The tumor volume was recorded every 2 days unless the mice displayed signs of prolonged distress, interference with normal movement or access to food and water, ulceration of the tumor, or when the tumor reached a size larger than 20 mm in diameter. On day 10, the tumors were harvested for photograph, tumor weight determination, H&E staining, Ki67 histochemistry staining, p62 histochemistry staining, LC3B histochemistry staining and TUNEL staining by One Step TUNEL Apoptosis Assay Kit.

#### *In vivo* survival observation

Hep-3B tumor-bearing BALB/c mice were generated through the injection of 2 ×10^6^ Hep-3B cells subcutaneously on the right side. Once the tumor volume reached approximately 100 mm^3^, the Hep-3B tumor-bearing mice were randomly divided into five groups (*n* = 10). On day 0, the mice were intravenously administered PBS, free PPE, free PPE+3MA, cL-P, cL-3 or asAN-P/3. These treatments were repeated every two days for a total of five administrations, with a dosage of 60.5 μg for PPE and 222 μg for 3MA. The tumor volume was calculated using the following standard formula: V = (tumor length) × (tumor width)^2^ /2. The tumor volume was recorded every 2 days unless the mice displayed signs of prolonged distress, interference with normal movement or access to food and water, ulceration of the tumor, or when the tumor reached a size larger than 20 mm in diameter until death in all groups except the asAN-P/3 group.

#### *In vivo* analysis of the biosafety of nanoformulations

Healthy BALB/c mice (*n* = 3) were administered PBS, free PPE, free PPE+3MA, cL-P, cL-3 or asAN-P/3 every two days for a total of five treatments (with dosages of 60.5 μg for PPE and 222 μg for 3MA). On day 10, blood samples were collected for blood biochemistry analysis, which included measurements of alanine transaminase (ALT), aspartate aminotransferase (AST), urine nitrogen (UREA), and creatinine (CRE-J) levels and blood routine analysis, which included measurements of red blood cells (RBC), hemoglobin (HGB), mean corpuscular hemoglobin (MCH), hematocrit (HCT), mean corpuscular volume (MCV), platelet (PLT), mean platelet volume (MPV), white blood cells (WBC), neutrophil and lymphocyte counts. Additionally, the main organs (heart, liver, spleen, lung and kidney) of the mice were harvested for histological examination using H&E staining.

### Statistical analysis

The data analysis was performed using GraphPad Prism version 10. Statistical analysis was conducted using unpaired t tests or paired t tests for comparisons between two groups, whereas one-way analysis of variance (ANOVA) was used for comparisons involving three or more groups. The half maximal inhibitory concentration (IC50) of the drugs and nanoformulations were determined by nonlinear regression (curve fit). Kaplan‒Meier survival curves were used to analyze animal survival, and statistical analysis was performed using the log-rank (Mantel‒Cox) test. A statistical significance threshold of *p* < 0.05 was applied. In the figures, the symbols * denote *p* < 0.05, ** denote *p* < 0.01, *** denote *p* < 0.001, **** denote *p* < 0.0001 and ns indicate no statistical significance.

## Results and Discussion

### Autophagy activation in HCC by PPE

First, Hep-3B cells were treated with different concentrations of an alternative to ELANE (PPE). The inhibitory effect of PPE on cell proliferation was observed to increase within the concentration range of 3.13 to 12.5 μg/mL. However, this effect reached a plateau at higher concentrations (25-400 μg/mL) (**Figure 2A**). To investigate this phenomenon, viability staining and apoptosis analysis were conducted. The results revealed that at high concentrations (400 μg/mL) of PPE, there was no significant increase in the number of propidium iodide (PI)-stained cells, yet a noticeable reduction in the number of calcein-stained cells was noted (**Figure S1**, Supporting Information). These results suggested that PPE could impede cell viability rather than induce cell death. This finding was further supported by the absence of a significant change in the apoptosis rate (**Figure S2**, Supporting Information). Additionally, the lactate dehydrogenase (LDH) release assay revealed no significant difference in the extracellular LDH levels of HCC cells treated with different concentrations of PPE (3.13-150 μg/mL) (**Figure S3**, Supporting Information). This observation indicated a lack of substantial cytotoxicity associated with PPE treatment. Overall, these findings demonstrate that PPE can inhibit HCC cell viability without promoting cell death.

To further investigate the mechanism underlying the observed inhibitory effect, an Edu assay was conducted. A notable reduction in Edu fluorescence within the PPE-treated group was observed, suggesting that PPE could effectively inhibit cell proliferation. Furthermore, cell cycle analysis demonstrated a significant increase in the proportion of cells in the G0/G1 phase. These findings indicate that PPE inhibits cell proliferation by arresting HCC cells in the quiescent phase (**Figure S4-S5**, Supporting Information). Collectively, these findings illustrate that the antitumor effect of PPE on Hep-3B cells primarily arises from the inhibition of cell proliferation rather than the induction of apoptosis.

**Figure 1:**
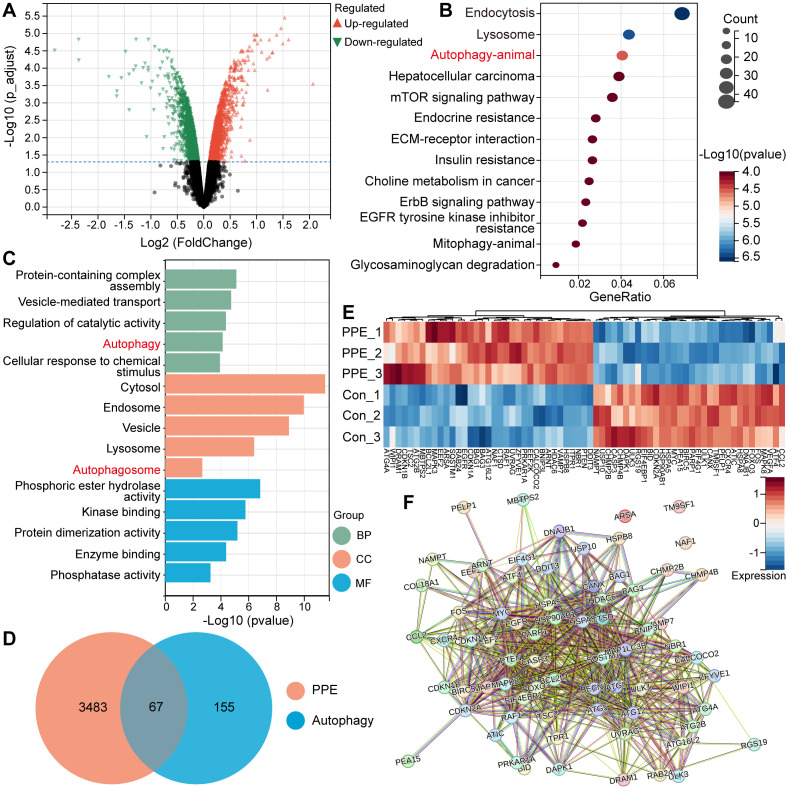
** PPE activated protective autophagy in HCC. A)** Volcano plot of differentially expressed genes in 3B cells treated with PPE compared with those in cells treated with PBS (control group). Red: upregulated genes; Green: downregulated genes. **B)** KEGG enrichment analysis of DEGs in 3B cells treated with PPE compared with those in the control group (identified genes). **C)** GO enrichment analysis of the identified genes. BP: Biological Process; CC: Cellular Component; MF: Molecular Function. **D)** Venn diagram of the identified overlapping DEGs in 3B cells treated with PPE and the autophagy-related gene data series from the Human Autophagy Database (HADb). **E)** Heatmap of the 67 autophagy-related DEGs between 3B cells treated with PPE and the control group. Blue: low expression level; Red: high expression level. **F)** PPI network analysis of the 67 autophagy-related DEGs in 3B cells treated with PPE.

To explore the functional mechanism of the limited therapeutic efficacy of PPE against hepatocellular carcinoma (HCC), transcriptome sequencing was performed. First, we identified 3550 differentially expressed genes (DEGs), including 1000 upregulated DEGs (**Figure 1A**). Gene Ontology (GO) and Kyoto Encyclopedia of Genes and Genomes (KEGG) analyses were subsequently used to perform biological enrichment of the identified upregulated DEGs.

KEGG analysis revealed that autophagy was the third most significant differential pathway. Notably, the first two differential pathways are tightly associated with the autophagy process (**Figure 1B**) 25. In addition, GO analysis revealed significant enrichment of autophagy in both the biological process (BP) and cellular component (CC) categories. In addition, the other enriched signals and functions were closely associated with autophagy (**Figure 1C**) 25, suggesting the activation of autophagic signals in the PPE group. These results revealed the same functional pathway, autophagy, in the PPE group.

Protective autophagy plays an important role in conferring drug resistance and promoting tumor development in HCC through the clearance of damaged or redundant peroxisomes, organelles and abnormal proteins 26. Based on these findings, all DEGs in the PPE group were merged with autophagy-related genes, and 67 overlapping DEGs related to autophagy **were identified (Figure 1D-E**) 27. Furthermore, protein‒protein interaction (PPI) network analysis revealed that nearly all of the identified genes interact with one another (**Figure 1F**), which affects the level of autophagy. These results indicate the important potential role that autophagy plays in PPE therapy.

**Figure 2:**
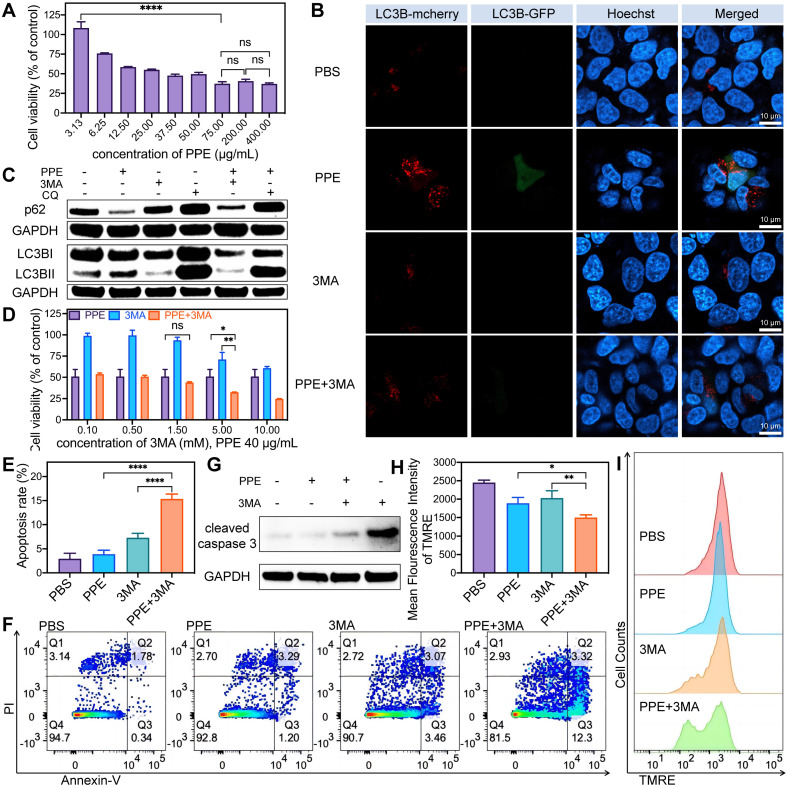
** PPE in combination with autophagy inhibitors synergistically induced cell apoptosis in HCC. A)** The cell viability of 3B cells after treatment with different concentrations of PPE was quantified by CCK8 assay. **B)** Fluorescence images of LC3B expression in 3B cells after different treatments. Red fluorescence from LC3B labeled with mCherry; green fluorescence from LC3B labeled with GFP; blue fluorescence represents nuclei stained with Hoechst 33342. The scale bar is 10 μm. **C)** Immunoblot analysis of the expression of the autophagy-related proteins p62, LC3B I and LC3B II in 3B cells after different treatments. **D) The c**ell viability of 3B cells after treatment with PPE (40 μg/mL), and different concentrations of 3MA were quantified by CCK8 assay. **E, F)** Apoptosis of 3B cells after different treatments was quantified by flow cytometry. **G)** Immunoblot analysis of the expression of the apoptosis-related protein cleaved caspase 3 in 3B cells after different treatments. **H, I)** Mitochondrial membrane potential of 3B cells after different treatments was quantified by flow cytometry. The data are expressed as mean ± SD. *p < 0.05, **p < 0.01, ***p < 0.001, ****p < 0.0001, ns: no statistical difference.

In light of these findings, Hep-3B cells were transfected with Ad-mCherry-GFP-LC3B to evaluate autophagy. The results revealed significant increases in both green (indicative of the early autophagy stage) and predominantly red (indicative of the full autophagy stage) fluorescence in cells treated with PPE. When 3MA was combined, both fluorescence signals were notably reduced, whereas when CQ was combined, they were significantly enhanced (**Figure 2B**, **Figure S6**, Supporting Information). These observations suggest that the autophagy induced by PPE can be suppressed by both the early inhibitor 3MA and the late inhibitor CQ. Specifically, 3MA impeded autophagosome formation, leading to a decreased level of LC3B. CQ hindered autophagolysosome formation and the degradation of metabolites, consequently resulting in the accumulation of LC3B 28. These findings indicate that PPE can induce complete autophagic flux in HCC cells.

In addition, the increase in LC3B II/LC3B I and the degradation of p62 are pivotal for assessing activated autophagy 29. The results revealed a substantial decrease in p62 and an increase in LC3B II/LC3B I, alongside elevated levels of Atg7 and Atg5 in cells treated with PPE 29. These changes were reversible upon treatment with 3MA or CQ (**Figure 2C**, **Figure S7-S8**, Supporting Information). These findings suggest that PPE can activate the autophagy signaling pathway, with the degradation of the autophagy substrate. Particularly, 3MA inhibited the expression of LC3B, whereas CQ increased the accumulation of LC3B and p62 by impeding their degradation 28. Based on the aforementioned findings, it can be concluded that PPE activates complete autophagic flux in HCC cells, involving the process from autophagosome formation to autophagolysosome maturation, with the subsequent degradation of cellular metabolites.

### 3MA enhanced the efficacy of PPE-mediated tumor therapy

To elucidate the role of PPE-induced autophagy in HCC, Hep-3B cells were treated with PPE along with the autophagy inhibitor 3MA. Cell viability was significantly inhibited in the combination group (**Figure 2D**). This observation suggested that inhibiting autophagy may increase the cytotoxicity of PPE in HCC. Subsequently, apoptosis analysis revealed a significantly elevated rate of apoptosis (**Figure 2E-2F**), especially early apoptosis, in the combination group (**Figure S9**, Supporting Information). In addition, the substantial activation of cleaved caspase 3 (**Figure 2G, Figure S10**, Supporting Information) and the significant reduction in the mitochondrial membrane potential (**Figure 2H-2I**) further supported the increase in cytotoxicity caused by the addition of 3MA 30,31. Collectively, these results indicated that the addition of an autophagy inhibitor could significantly promote cell death in HCC, highlighting the protective role of autophagy in PPE treatment.

**Figure 3:**
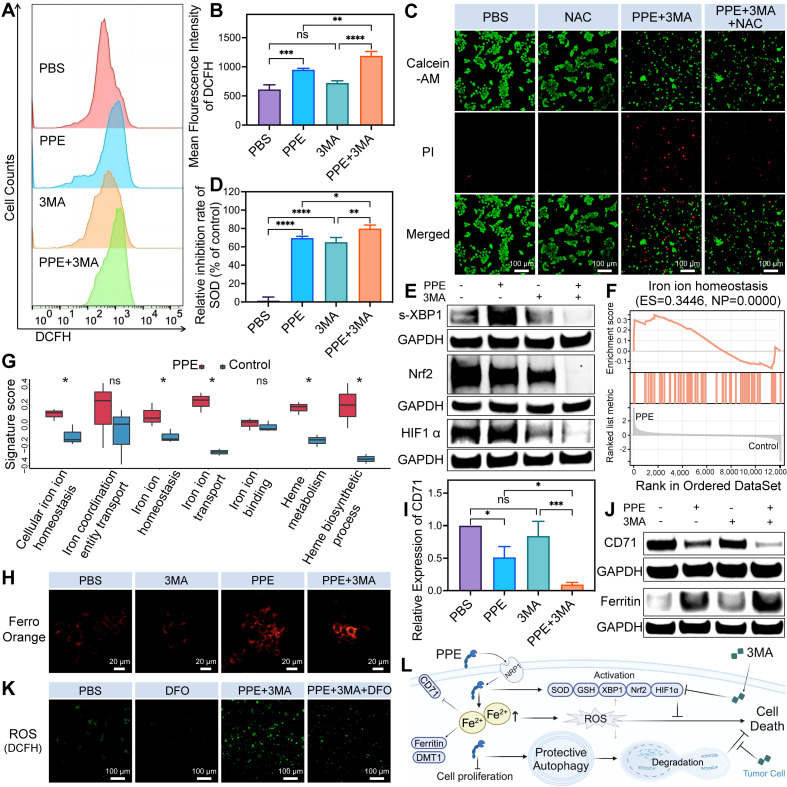
** 3MA enhanced PPE-mediated iron-related ROS-mediated cell apoptosis in HCC. A, B)** ROS levels in 3B cells after different treatments were quantified by flow cytometry. **C)** The cytotoxicity of 3B cells after different treatments was assessed by calcein-AM/PI costaining assay. Green fluorescence from calcein-AM; red fluorescence from PI. The scale bar is 100 μm. **D)** SOD inhibition rate evaluation of 3B cells after different treatments. **E)** Immunoblot analysis of the expression of the antioxidant nuclear transcription factors s-XBP1, Nrf2 and HIF1α in 3B cells after different treatments. **F)** Representative iron metabolism-related pathways enriched with the identified genes were determined by GSEA. **G)** Iron metabolism-related pathways enriched with the identified genes were evaluated by GSVA enrichment scores. **H)** Fluorescence images of ferrous ion in 3B cells after different treatments. Orange fluorescence from FerroOrange. The scale bar is 20 μm. **I)** Expression of CD71 in 3B cells after different treatments was quantified by immunoblot analysis. **J)** Immunoblot analysis of the expression of the iron metabolism-related proteins CD71 and ferritin in 3B cells after different treatments. **K)** Fluorescence images of ROS in 3B cells after different treatments. Green fluorescence from DCFH. The scale bar is 100 μm. **L)** Schematic illustration of the functional mechanism by which 3MA enhances the therapeutic efficacy of PEE in HCC. The data are expressed as mean ± SD. *p < 0.05, **p < 0.01, ***p < 0.001, ****p < 0.0001, ns: no statistical difference.

Subsequently, we investigated the underlying mechanism of the combination treatment in HCC. Prior studies have shown that PPE can induce cell death by disrupting mitochondria and generating ROS through CD95 cleavage 11. Our assessment of intracellular ROS levels revealed a significant increase in the PPE group, with no discernible change in the 3MA group. Interestingly, the combination group presented markedly elevated levels of ROS compared with the individual treatment groups (**Figure 3A-3B**), indicating substantial induction of oxidative stress in HCC in the combination group.

Notably, tumor cells have the capacity to counteract ROS through the activation of protective autophagy and antioxidant pathways. These pathways include a variety of antioxidant effectors, such as superoxide dismutase (SOD) 32, glutathione (GSH) 33, nuclear factor erythroid 2-related factor 2 (Nrf2) 34, hypoxia-inducible factor 1α (HIF1α) and the spliced activated form of unfolded protein response transcription factor X-box-binding protein 1 (s-XBP1) 35,36. These effectors effectively remove ROS, maintain cellular homeostasis, inhibit cell death, and contribute to drug resistance.

Therefore, we assessed the changes in antioxidant signals. The results of the SOD inhibition rate and GSH level revealed increased consumption of SOD and GSH in both the PPE and 3MA groups, while the combination group presented the most significant depletion (**Figure 3D**, **Figure S11**, Supporting Information). In light of these previous findings, the consumption of SOD and GSH inhibited cell death in the PPE group. However, it increased the degree of cell death in the combination group. This occurred because the consumption of SOD and GSH prevents ROS-mediated cell death, whereas the exhaustion (excessive consumption, high degree of consumption) of SOD and GSH promotes cell apoptosis 37,38.

Further analysis revealed substantial upregulation of s-XBP1 and HIF1α in the PPE group, with no significant change in Nrf2. Notably, the addition of 3MA led to significant suppression of these three factors (**Figure 3E**, **Figure S12**, Supporting Information). This observation suggests that HIF1α and s-XBP1, rather than Nrf2, function as the primary antioxidants in the PPE group. This is attributed to the suppressive effects of PPE and XBP1 on the expression of Nrf2 39,40, resulting in insufficient Nrf2-mediated antioxidant capacity and the activation of alternative antioxidant factors 41. These findings illustrate that although PPE increases intracellular oxidative stress by generating ROS, tumor cells are capable of mitigating stress and maintaining homeostasis through the activation of antioxidant pathways.

However, this limitation could be reversed by 3MA. It inhibits the activity of SOD and GSH 42, as well as the expression of HIF1α 43, Nrf2 and XBP1 44,45, to promote oxidative stress-mediated cell death. In light of these findings, we investigated the important role of ROS in cancer cell death by utilizing N-acetylcysteine (NAC) to remove ROS. Notably, we observed a significant increase in cell death in the combination group, which was reversed by NAC (**Figure 3C**). These findings indicate that 3MA has the potential to enhance the therapeutic efficacy of PPE through oxidative stress *in vitro*.

Building upon these findings, we investigated the origins of the ROS induced by the combination treatment in HCC. Notably, the expression of CD95 is low in HCC, especially in the late stage, with a silenced function in HCC (**Figure S13**, Supporting Information) 46, suggesting the possibility of alternative mechanisms contributing to ROS generation. The liver serves as the predominant iron pool in the body owing to its enormous storage of iron and proteins containing Fe-S, rendering it highly reactive toward ROS 47. As a result, the impact of iron metabolism on oxidative stress in HCC has garnered increasing attention in recent years 48. In addition, ELNAE has been shown to regulate breast cancer progression by degrading insulin receptor substrate 1 (IRS1) 49, the loss of which leads to increased iron levels in the liver 50. We observed similar degradation of IRS1 in cells treated with PPE (**Figure S14**, Supporting Information).

Therefore, iron metabolism may contribute to the efficacy of PPE-mediated therapy in HCC. Therefore, we obtained datasets of genes related to iron metabolism 51. We subsequently performed gene set enrichment analysis (GSEA) and gene set variation analysis (GSVA) for the upregulated DEGs in the PPE group. GSEA revealed that PPE significantly upregulated iron ion homeostasis (**Figure 3F**). Moreover, GSVA signature score analysis revealed five significantly upregulated iron metabolism-related pathways in the PPE group, with distinct increasing trends in the other two pathways (**Figure 3G**). These results indicate that iron metabolism has an important potential role in PPE therapy.

The ROS produced by iron metabolism primarily originate from ferrous ions 48. Hence, we assessed the intracellular ferrous ion content and observed a significant increase in cells treated with PPE, which was further elevated when PPE was combined with 3MA (**Figure 3H**). Notably, iron overload downregulates transferrin receptor 1 (TfR1, also known as CD71) and divalent metal transporter 1 (DMT1) through negative feedback regulation while upregulating ferritin to bind excess iron 52-54, thereby reducing the level of intracellular iron.

Consequently, we detected a significant reduction in the expression of CD71 and an increase in the expression of ferritin in cells treated with PPE. When 3MA was combined with 3MA, the expression of CD71 significantly decreased, whereas the expression of ferritin tended to increase (**Figure 3I-3J**, **Figure S15**, Supporting Information). Nevertheless, there was no discernible change in the expression of DMT1 across the different groups (**Figure S16**, Supporting Information). This is attributed to the fact that the downregulation of DMT1 is the consequence of prolonged iron overload rather than short-term variation 55. These findings suggest that PPE can induce iron overload in HCC, with a higher level in the combination group.

To investigate the important role of iron metabolism in the induction of oxidative stress in HCC, deferoxamine (DFO) was subsequently utilized to eliminate excess ferrous ions. We observed a noticeably elevated level of ROS in the combination group, which was markedly reduced by DFO (**Figure 3K**). This observation indicated the crucial role of iron in ROS generation in the combination group. Overall, the aforementioned findings illustrate that PPE can induce oxidative stress by regulating iron metabolism in HCC, with an amplified effect in combination with 3MA. Notably, the regulatory effect of therapy on oxidative stress induced by iron metabolism is not a unilateral outcome but rather the result of a comprehensive interplay involving mitochondrial destruction 56, autophagy 54, iron-related protein metabolism and IRS1 degradation 50,57.

In summary, although PPE, an alternative to ELANE, inhibits proliferation and induces oxidative stress through iron overload in HCC, its efficacy is constrained by the cellular activation of protective autophagy and antioxidant pathways, which protects HCC cells from death. Conversely, the addition of 3MA inhibits not only protective autophagy but also antioxidant pathways, thereby synergistically sensitizing the toxicity of PPE-mediated therapy in HCC (**Figure 3L**).

### Preparation and characterization of autophagy inhibitor-sensitized artificially activated neutrophils

**Figure 4:**
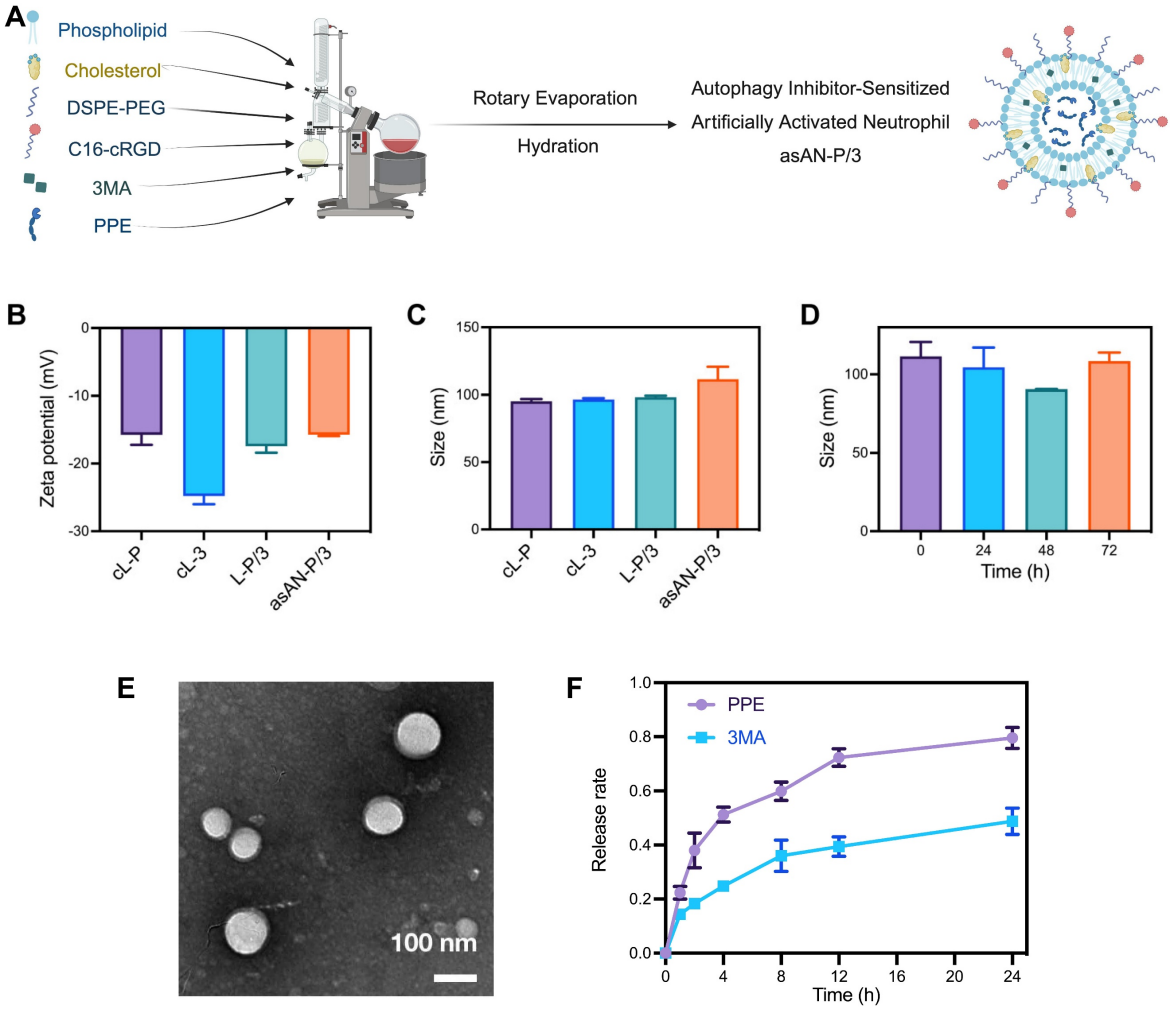
** Preparation and characteristics of the asAN-P/3. A)** Diagram of asAN-P/3 preparation by the rotary evaporation method. **B)** The zeta potential of different nanomedicines. **C)** The sizes of different nanomedicines. **D)** Size of asAN-P/3 at different times. **E)** Transmission electron microscopy (TEM) images of asAN-P/3 in PBS. The scale bar is 100 nm. **F)** Release rates of different drugs loaded in asAN-P/3 at different time points. cL-P: cRGD-modified liposomes loaded with PPE; cL-3: cRGD-modified liposomes loaded with 3MA; L-P/3: liposomes loaded with PPE and 3MA; asAN-P/3: autophagy inhibitor-sensitized artificially activated neutrophils. The data are expressed as mean ± SD.

The alternative to ELANE, PPE, can be inactivated by serum (**Figure S17**, Supporting Information) owing to the natural protease inhibitors in the blood and the consumption by degrading extracellular connective tissue 14-16. Moreover, there is a high demand for cells from patients receiving neutrophil-based carriers, and their targeting ability depends on the level of inflammatory chemotaxis signaling in the tumor 6. To solve the above problems, we prepared a targeted liposomal nanoplatform with cRGD via the rotary evaporation method (**Figure 4A**). Among them, phospholipid, cholesterol and DSPE-PEG consisted of framework nanoparticles in asAN-P/3. C16-cRGD was utilized as the targeting peptide modified on asAN-P/3 to target HCC tumor cells. In addition, PPE and 3MA loaded in asAN-P/3 were delivered through the cRGD-modified nanoplatform into HCC cells as effective combination drugs, synergistically increasing tumor cell death.

To optimize the component ratios, 3MA loading ratios were first assessed for nanoparticle cytotoxicity, revealing a dose-dependent increase that plateaued at 3:1 (3MA:PPE) (**Figure S18**, Supporting Information). The cRGD ratios were subsequently evaluated for their ability to target cells, and the results revealed enhanced uptake until saturation at 4:1 (cRGD:PPE) (**Figure S19**, Supporting Information). Based on these results, the therapeutic nanoparticle asAN-P/3 was synthesized for further study.

The density of cRGD in asAN-P/3 was approximately 0.144 g/cm^3^ (**Figure S20**, **Table S1**, Supporting Information), which was detected by fluorescence labeling and absorption spectroscopy. The loading and encapsulation efficiencies for PPE were determined to be 2.16% and 60.5%, respectively, whereas those for 3MA were 7.93% and 74.0%, respectively. The hydrodynamic diameter and surface zeta potential of asAN-P/3 were 111.53 ± 9.17 nm and -15.73 ± 0.21 mV, respectively, which were similar to those of other control nanomedicines (**Figure 4B-4C**).

The asAN-P/3 exhibited typical vesicle structures with a phospholipid bilayer coating. The dry diameter of these particles was approximately 100 nm, which was smaller than the hydrated diameter due to particle contraction (**Figure 4C, Figure 4E**). Moreover, the diameter of asAN-P/3 remained constant in phosphate-buffered saline (PBS) at pH 7.4 over a period from 0 to 72 h (**Figure 4D**), demonstrating excellent stability under neutral conditions. We also detected the hydrated diameter of asAN-P/3 in plasma on different days. The results showed that its diameter is slightly larger than that in water, which may be a result of surface protein absorption of asAN-P/3 in plasma. In addition, the diameter of asAN-P/3 did not obviously change, indicating that it remained stable in the plasma (**Figure S21**, Supporting Information).

To evaluate the release kinetics of 3MA and PPE, asAN-P/3 was encapsulated within a dialysis bag with a molecular weight cutoff (MWCO) of 300 kDa and subsequently immersed in PBS containing Tween 80 (0.1%, w/v) at 37°C under oscillation conditions. The results revealed rapid release of PPE, with 79.6% released within the initial 24 h. Compared with free PPE, the released PPE retained 94.79 ± 0.86% of the enzymatic activity, indicating preserved functionality postnanoformulation (**Figure S22**, Supporting Information). In contrast, 3MA exhibited a sustained release profile, with only 49.2% released within the same period (**Figure 4F**). This difference in release rates may be attributed to the differing solubility properties, where PPE is more hydrophilic and 3MA is more hydrophobic.

### *In vitro* evaluation of the tumor targeting and therapeutic potential of asAN-P/3

**Figure 5:**
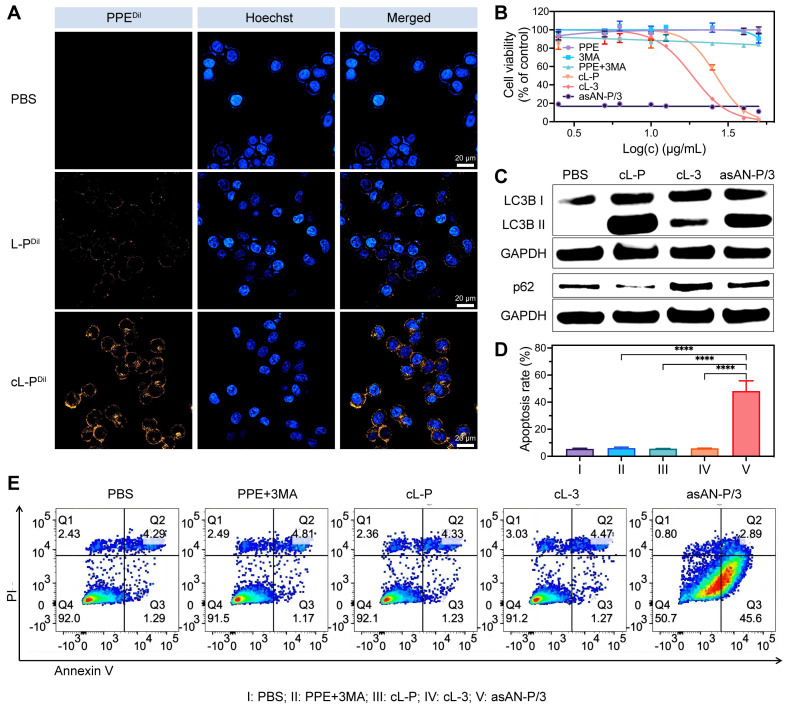
** Enhanced targeting ability and therapeutic efficacy of the asAN-P/3 *in vitro*. A)** Confocal laser scanning microscopy (CLSM) images of 3B cells incubated with PBS, L-P or cL-P for 3 h. Orange fluorescence from PPE labeled with Dil; blue fluorescence indicates nuclei stained with Hoechst 33342. The scale bar is 20 μm. **B)** Concentration-dependent cytotoxicity evaluation of 3B cells after different treatments. The concentration of the nanomedicines was dependent on PPE, and the concentration of 3MA was 3.67 times greater than that of PPE. **C)** Immunoblot analysis of the expression of the autophagy-related proteins LC3B I, LC3B II and p62 in 3B cells after different treatments. **D, E)** Apoptosis of 3B cells after different treatments was quantified by flow cytometry. I: **PBS**; II: **PPE + 3MA**; III: **cL-P;** IV: **cL-3;** V: **asAN-P/3.** L-P: liposomes loaded with PPE; cL-P: cRGD-modified liposomes loaded with PPE; cL-3: cRGD-modified liposomes loaded with 3MA; asAN-P/3: autophagy inhibitor-sensitized artificially activated neutrophils. The data are expressed as mean ± SD. *p < 0.05, **p < 0.01, ***p < 0.001, ****p < 0.0001.

To investigate the intracellular delivery capability and therapeutic efficacy of the autophagy inhibitor-sensitized artificially activated neutrophils (asAN-P/3) in HCC *in vitro*, we initially treated Hep-3B cells with Dil-labeled nanomedicines. The cL-P group exhibited the most pronounced fluorescence signal (**Figure 5A**), indicating enhanced cellular uptake ability. To assess the therapeutic efficacy of asAN-P/3 *in vitro*, Hep-3B cells were subsequently treated with different drugs. We observed the highest level of toxicity in the asAN-P/3 group (**Figure 5B**), with a significantly reduced IC50 of 117.6 ng/mL (**Figure S23**, Supporting Information). Moreover, asAN-P/3 significantly increased the apoptosis rate of HCC **cells (Figure 5D-5E**), especially early apoptosis (**Figure S24**, Supporting Information).

Furthermore, an analysis of autophagy-related proteins revealed that the cL-P group presented increased conversion of LC3B, with substantial degradation of p62. These effects were reversed by 3MA in the asAN-P/3 group, suggesting that 3MA inhibited the increase in autophagy induced by PPE. Notably, the cL-3 group exhibited marked upregulation of p62 alongside a moderate increase in LC3B expression (**Figure 5C, Figure S25**, Supporting Information), indicating that the inhibitory effect on autophagy caused by 3MA could cumulatively disrupt cellular homeostasis, thereby slightly activating autophagy when the inhibitory effect was eliminated 58. Collectively, these findings demonstrate that asAN-P/3 not only facilitates efficient intracellular delivery but also greatly enhances cytotoxicity against HCC by modulating autophagy *in vitro.*

### *In vivo* evaluation of the tumor targeting and therapeutic potential of asAN-P/3

**Figure 6:**
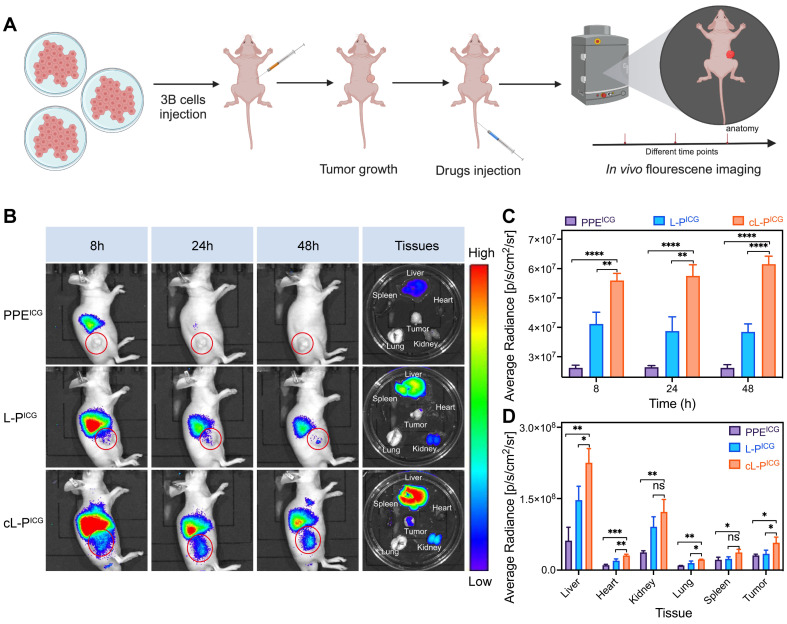
** Enhanced tumor-targeting ability *in vivo*. A)** Schematic illustration of the experimental design of *in vivo* fluorescence imaging in mice. **B, C, D)** *In vivo* targeting ability to tumor tissues of mice after injection of PPE^ICG^, L-P^ICG^ or cL-P^ICG^ at different time points was quantified by *in vivo* fluorescence imaging, and *ex vivo* accumulation in major organs and tumor tissues of mice was quantified by* ex vivo* fluorescence imaging. The red circle highlights the tumor area. L-P: liposomes loaded with PPE; cL-P: cRGD-modified liposomes loaded with PPE. The data are expressed as mean ± SD. *p < 0.05, **p < 0.01, ***p < 0.001, ****p < 0.0001, ns: no statistical difference.

Based on the excellent cellular uptake ability of cL-P *in vitro*, we investigated the targeting ability by biodistribution of cL-P *in vivo*, as depicted in **Figure 6A**. At 8, 24, and 48 hours post intravenous injection, a significant increase in tumor accumulation was observed within the cL-P group (**Figure 6B-6C**). These observations suggested that nanomization and cRGD modification significantly enhanced the targeting specificity.

The major organs and tumor tissues from the experimental mice were subsequently collected for further biodistribution analysis. The results revealed a significant increase in fluorescence within the tumor tissues of the cL-P group, further indicating the targeting ability of nanoliposomes modified with cRGD (**Figure 6B**, **Figure 6D**). Notably, notable accumulation of PPE was observed in blood-rich organs, particularly the liver (**Figure 6B, Figure 6D**). However, as shown in **Figure 6B**, the fluorescence intensity in the liver clearly decreased over time, which was attributed to the gradual clearance of the nanomedicine by systemic circulation mechanisms 59.

**Figure 7:**
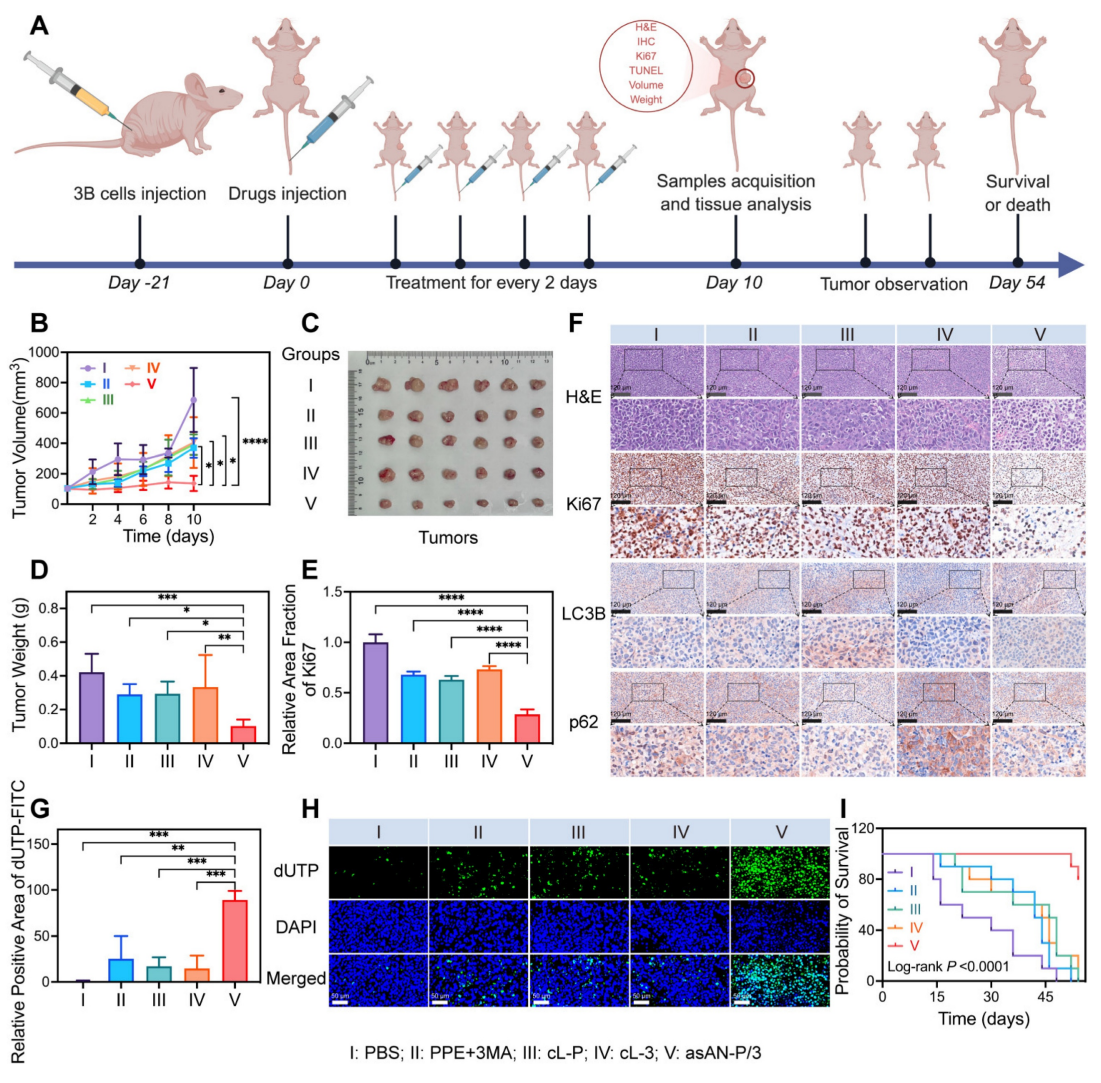
** Enhanced therapeutic efficacy of asAN-P/3 by the regulation of autophagy* in vivo*. A)** Schematic illustration of the experimental design for determining treatment efficacy *in vivo in mice*. **B)** Growth curves of tumors from mice after different treatments. **C)** Photograph of *ex vivo* tumors from mice after different treatments on day 10.** D)** Tumor weights of *ex vivo* tumors from mice after different treatments on day 10.** E)** Quantification of Ki67 immunohistochemistry analysis. **F)** H&E staining and immunohistochemistry of Ki67, LC3B and p62 in tumors from mice after different treatments on day 10. The scale bar is 120 μm. **G)** Quantification of TUNEL apoptosis analysis. **H)** Fluorescence images of TUNEL apoptosis analysis of tumors from mice after different treatments on day 10. Green fluorescence from dUTP labeled with FITC; blue fluorescence from DAPI. The scale bar is 50 μm. **I)** Survival curves of the mice after different treatments. I: **PBS**; II: **PPE + 3MA**; III: **cL-P;** IV: **cL-3;** V: **asAN-P/3.** cL-P: cRGD-modified liposomes loaded with PPE; cL-3: cRGD-modified liposomes loaded with 3MA; asAN-P/3: autophagy inhibitor-sensitized artificially activated neutrophils. The data are expressed as mean ± SD. *p < 0.05, **p < 0.01, ***p < 0.001, ****p < 0.0001.

Encouraged by the excellent targeting ability of the asAN-P/3, we investigated the therapeutic efficacy of asAN-P/3 *in vivo*. The mice were treated with PBS, free PPE, free PPE+3MA, cL-P, cL-3 or asAN-P/3, as shown in **Figure 7A**. Tumor growth analysis revealed no significant inhibition in the free PPE group compared with the PBS control group, whereas the free PPE+3MA, cL-P, and cL-3 groups exhibited moderate suppression, indicating enhanced therapeutic efficacy through nanoformulation. Notably, the asAN-P/3 group achieved the highest tumor inhibition rate (80.09 ± 7.35%, **Figure 7B-7C, Figure S26A-S26B, Supporting Information**), with tumor weight measurements corroborating this pronounced suppression (**Figure 7D, Figure S26C, Supporting Information**). These findings highlight the substantial suppression in the asAN-P/3 group.

The H&E staining results revealed that, compared with those in the other groups, the tumors in the asAN-P/3 group presented the highest levels of tumor cell apoptosis and necrosis (**Figure 7F**). These findings indicate that asAN-P/3 possesses potent antitumor capabilities and effectively suppresses tumor progression. Based on these observations, we assessed the proliferation ability and degree of apoptosis of tumor tissues by Ki67 immunohistochemistry and TUNEL assays 60,61. The Ki67 immunohistochemistry results demonstrated that, compared with the other groups, the asAN-P/3 group presented the greatest inhibition of tumor proliferation (**Figure 7E-7F**). Additionally, the TUNEL assay results indicated that, in comparison with the PBS group, the free PPE+3MA, cL-P and cL-3 groups exhibited negligible apoptosis, whereas the asAN-P/3 group exhibited significantly increased apoptosis, both in terms of the proportion of apoptotic cells (**Figure 7G-7H**) and the apoptotic area (**Figure S27**, Supporting Information). These findings demonstrate that asAN-P/3 can effectively inhibit tumor cell proliferation while significantly promoting tumor apoptosis, thereby synergistically enhancing the therapeutic efficacy against tumors.

Additionally, immunohistochemical staining of LC3B and p62 was performed on the tumor tissues to elucidate the underlying mechanism *in vivo*. Compared with the PBS group, the group treated with free PPE+3MA did not show obvious regulatory effects, potentially due to the lack of specific targeting. Compared with the cL-P group, the asAN-P/3 group presented significantly downregulated LC3B and upregulated p62, indicating that 3MA could effectively inhibit the autophagy induced by PPE (**Figure 7F, Figure S28**, Supporting Information).

Inspired by the excellent short-term antitumor ability of the asAN-P/3, we monitored the long-term survival rates of the asAN-P/3-treated mice. Survival curves revealed slight improvement in the free PPE group compared with the PBS control group, with moderate enhancement in the free PPE+3MA, cL-P, and cL-3 groups. Notably, asAN-P/3 achieved the highest survival rate, demonstrating its enhanced efficacy in prolonging survival (**Figure 7I, Figure S29**, Supporting Information). Collectively, these findings illustrate that the autophagy inhibitor-sensitized artificially activated neutrophils (asAN-P/3) displays precise HCC targeting ability, outstanding antitumor efficacy and prolonged survival ability through the regulation of autophagy *in vivo*.

### *In vivo* biocompatibility of asAN-P/3

**Figure 8:**
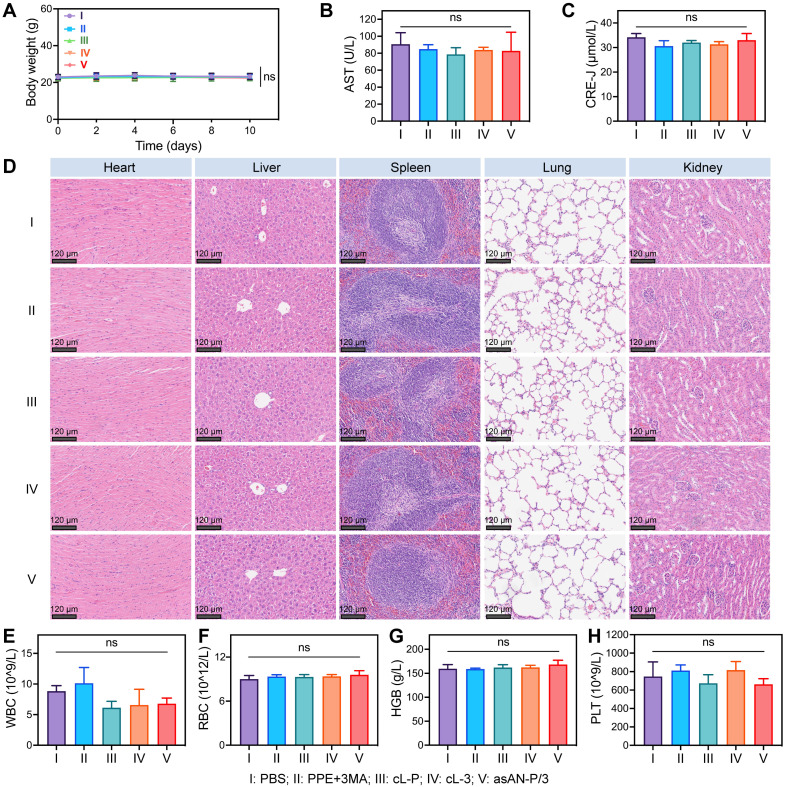
** Biosafety of the asAN-P/3 *in vivo*. A)** Curves of the body weights of the mice after different treatments at different time points. Hepatic function and renal function were evaluated by the quantification of **B)** glutamic oxaloacetic transaminase (AST) and **C)** creatinine (CRE-J) levels. **D)** H&E staining of major organs from mice after different treatments on day 10. The scale bar is 120 μm. **E)** The inflammatory state was evaluated by quantifying white blood cells (WBC) from the mice after different treatments on day 10. The hemolysis reaction was evaluated by the quantification of **F)** red blood cells (RBC) and** G)** hemoglobin (HGB). **H)** Coagulation function was evaluated by the quantification of platelets (PLT) from mice after different treatments on day 10. I: **PBS**; II: **PPE + 3MA**; III: **cL-P;** IV: **cL-3;** V: **asAN-P/3.** cL-P: cRGD-modified liposomes loaded with PPE; cL-3: cRGD-modified liposomes loaded with 3MA; asAN-P/3: autophagy inhibitor-sensitized artificially activated neutrophils. The data are expressed as mean ± SD. ns: no statistical difference.

The *in vivo* biocompatibility of the autophagy inhibitor-sensitized artificially activated neutrophils (asAN-P/3) was further assessed. The body weights of the mice were monitored throughout the treatment period. The results revealed no significant weight loss in any of the experimental groups (**Figure 8A, Figure S30A, Supporting Information**). Blood biochemistry revealed no statistically significant differences in aspartate transaminase (AST) or alanine transaminase (ALT) levels between the asAN-P/3 group and the control group (**Figure 8B**, **Figure S30B-C, S31A**, Supporting Information), indicating preserved hepatic function 62. Similarly, the renal markers creatinine (CRE-J) and urea (UREA) showed no alterations across the groups (**Figure 8C**, **Figure S30D-E, S31B**, Supporting Information) 62, confirming systemic safety. Histological examination of the liver, heart, lungs, spleen and kidneys revealed no signs of tissue toxicity in any of the experimental groups (**Figure 8D**). Additionally, key hematological parameters (white blood cell count, red blood cells, hemoglobin, coagulation) were not significantly different among the experimental groups (**Figure 8E-8H, Figure S32-S33,** Supporting Information), indicating normal hematological function. Nonetheless, nontargeting free PPE may be taken up by normal cells, causing a mild inflammatory response that in turn causes an increase in the number of neutrophils in the free PPE+3MA group and a decrease in the number of lymphocytes in the free PPE group 63, which was not observed in the nanomedicine groups (**Figure S32E, S33B**, Supporting Information).

In summary, these findings suggest that the autophagy inhibitor-sensitized artificially activated neutrophils (asAN-P/3) exhibit no significant systemic toxicity, indicating its potential as a promising therapeutic agent with superior biocompatibility for HCC in the future.

## Conclusion

**Scheme 2:**
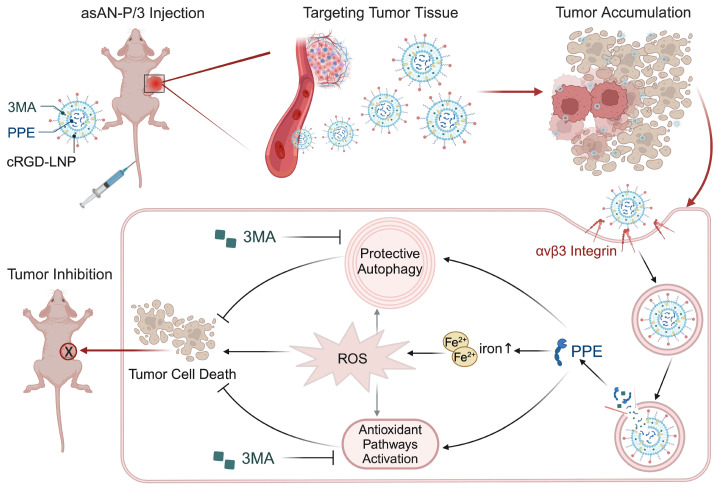
** Schematic illustration of the autophagy inhibitor-sensitized artificially activated neutrophils (asAN-P/3) for HCC therapy.** The alternative to neutrophil elastase, PPE, and the autophagy inhibitor 3MA are encapsulated in cRGD-modified liposomes to synthesize asAN-P/3, which is targeted for transport through the blood circulation and accumulated in tumor tissue. After entering tumor cells through the interaction of cRGD with αvβ3 integrin, PPE and 3MA are released. PPE destroys tumor cells by generating iron-dependent ROS, and 3MA inhibits both protective autophagy and antioxidant pathways to sensitize PPE-mediated cell destruction. They synergistically promote tumor cell death. This autophagy inhibitor-sensitized artificially activated neutrophils (asAN-P/3) significantly enhanced the targeting ability and therapeutic efficacy in HCC.

In this work, we discovered the limited therapeutic efficacy of the activated neutrophil-killing effector mimicker PPE in HCC. It activates protective autophagy and antioxidative signals to protect tumor cells from death, which limits its therapeutic potential. Importantly, the incorporation of 3MA, an autophagy inhibitor, sensitizes PPE by enhancing the iron-related ROS-mediated destruction of cells induced by PPE. Subsequently, the autophagy inhibitor-sensitized artificially activated neutrophils (asAN-P/3) were prepared to target HCC and improve therapeutic efficacy, overcoming the constraints of neutrophil-based and artificial neutrophil-related antitumor therapy (**Scheme 2**). *In vitro* and *in vivo* evidence has revealed the precise targeting ability, excellent therapeutic efficacy, prolonged survival and favorable biocompatibility of asAN-P/3. Our study contributes valuable insights for the advancement and application of neutrophil-related therapeutic strategies in oncology in the future.

## Supplementary Material

Supplementary figures.

## Abbreviations

ELANE: neutrophil elastase
PPE: porcine pancreatic elastase
HCC: hepatocellular carcinoma
ROS: reactive oxygen species
3MA: 3-methyladenine
cRGD: cyclic arginine glycine aspartate peptide
PI3K: phospholipase-like III enzymes
PBS: phosphate buffer solution
NAC: N-acetylcysteine
CCK8: Cell Counting Kit-8
TMRE: tetramethylrhodamine ethyl ester
CQ: chloroquine
DFO: deferoxamine
HADb: Human Autophagy Database
LDH: lactate dehydrogenase
IRS1: insulin receptor substrate 1
SOD: superoxide dismutase
GSH: glutathione
GSSG: oxidized glutathione
GO: gene ontology
KEGG: Kyoto Encyclopedia of Genes and Genomes
BP: biological process
CC: cellular component
MF: molecular function
PPI: protein‒protein interaction
GSEA: gene set enrichment analysis
GSVA: gene set variation analysis
H&E: Hematoxylin‒eosin
ALT: alanine transaminase
AST: aspartate aminotransferase
UREA: urine nitrogen
CRE‒J: creatinine
RBC: red blood cells
HGB: hemoglobin
PLT: platelets
MPV: mean platelet volume
WBC: white blood cells
ANOVA: one-way analysis of variance
PI: propidium iodide
DEGs: differentially expressed genes
Nrf2: nuclear factor erythroid 2-related factor 2
HIF1α: hypoxia-inducible factor 1α
s-XBP1: spliced X-box-binding protein 1
TfR1(same as CD71): transferrin receptor 1
DMT1: divalent metal transporter 1
MWCO: molecular weight cut-off
TEM: transmission electron microscopy
CLSM: confocal laser scanning microscopy
TUNEL: TdT-mediated dUTP nick end labeling
