# Supplementary material for: Autophagy inhibitor-sensitized artificially activated neutrophils against hepatocellular carcinoma
Source: Theranostics. 2025 Jun 18;15(14):7197–218. doi: 10.7150/thno.106404 (PMC12204081; doi:10.7150/thno.106404)
Supplement: Supplementary file 1 — Supplementary figures. [file thnov15p7197s1.pdf]

Supporting Information

Autophagy inhibitor-sensitized artificially activated neutrophils against hepatocellular carcinoma

Caixia Yang[1, 2, 3, 4, 5#], *Huang Yang*[1, 6#], *Zhengwei Mao*[1, 6*], *Weilin Wang*[1,2, 3, 4, 5*], *Yuan Ding*[1,2, 3, 4, 5*]

[1] Department of Hepatobiliary and Pancreatic Surgery, the Second Affiliated Hospital, Zhejiang University School of Medicine, Hangzhou, Zhejiang 310009, China

[2] Key Laboratory of Precision Diagnosis and Treatment for Hepatobiliary and Pancreatic Tumor of Zhejiang Province, Hangzhou, Zhejiang 310009, China

[3] Research Center of Diagnosis and Treatment Technology for Hepatocellular Carcinoma of Zhejiang Province, Hangzhou, Zhejiang 310009, China

[4] Center for Medical Research and Innovation in Digestive System Tumors, Ministry of Education, Hangzhou, Zhejiang 310009, China

[5] Cancer Center, Zhejiang University, Hangzhou, Zhejiang 310058, China

[6] MOE Key Laboratory of Macromolecular Synthesis and Functionalization, Department of Polymer Science and Engineering, Zhejiang University, Hangzhou, Zhejiang, China

[#] Contributed equally.

[*] Corresponding author.

E-mail address: dingyuan@zju.edu.cn (Yuan Ding); wam@zju.edu.cn (Weilin Wang)

zwmao@zju.edu.cn (Zhengwei Mao)

Telephone and fax numbers: Tel: +86057187783820; Fax: +86057187068001

OCIRD: 0000-0001-9432-2649 (Weilin Wang).

Supporting Information Figures

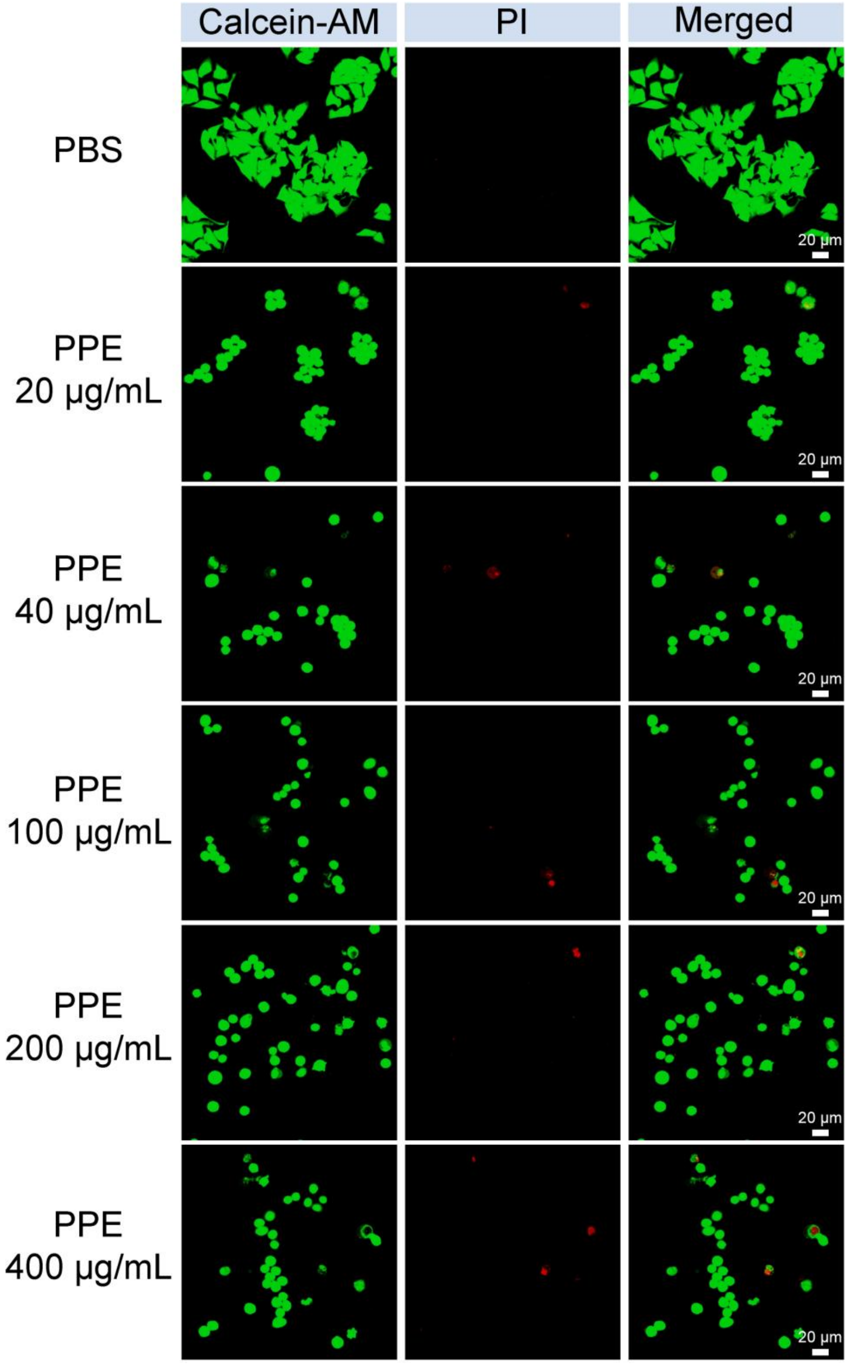

Figure S1. The cytotoxicity of 3B cells after treatment with different concentrations of PPE was assessed by calcein-AM/PI co-staining assay. Green fluorescence from calcein-AM; red fluorescence from PI. The scale bar is 20 μm.

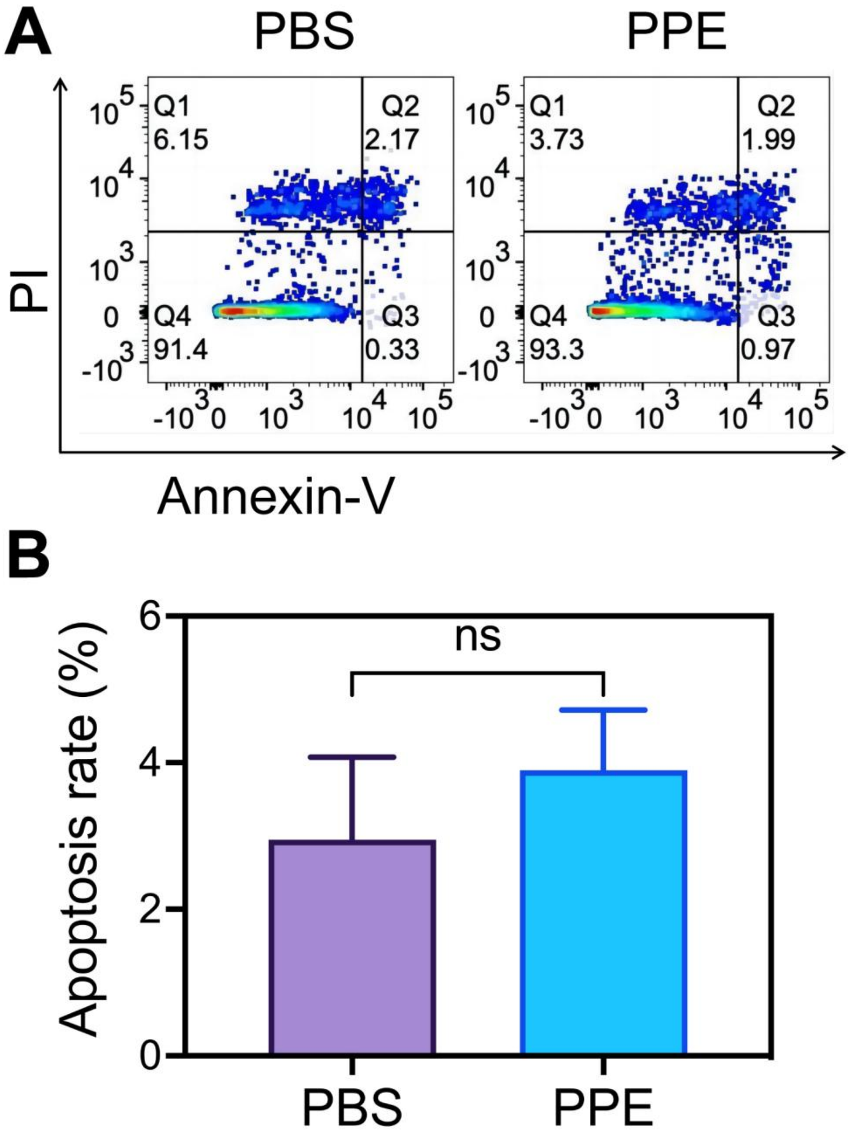

Figure S2. A, B) Apoptosis of 3B cells after treatment with PBS or PPE (40 μg/mL) was quantified by flow cytometry. The data are expressed as mean ± SD. ns: no statistical difference.

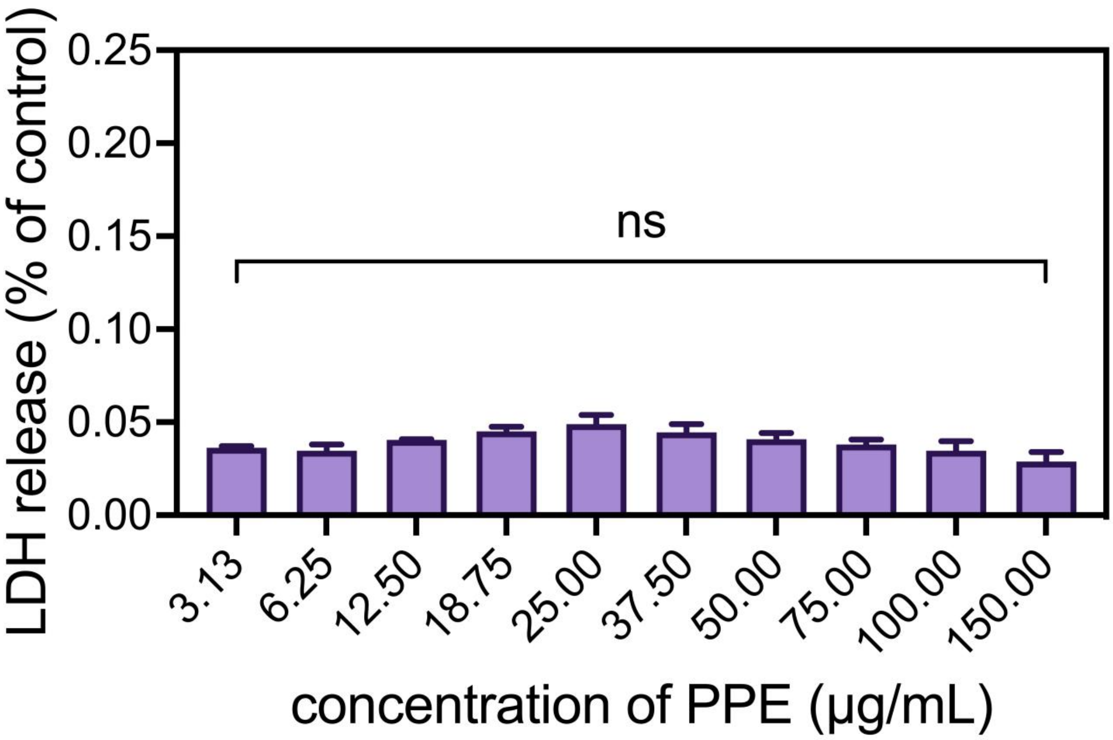

Figure S3. The cytotoxicity of 3B cells after treatment with different concentrations of PPE was quantified by LDH release. The data are expressed as mean ± SD. ns: no statistical difference.

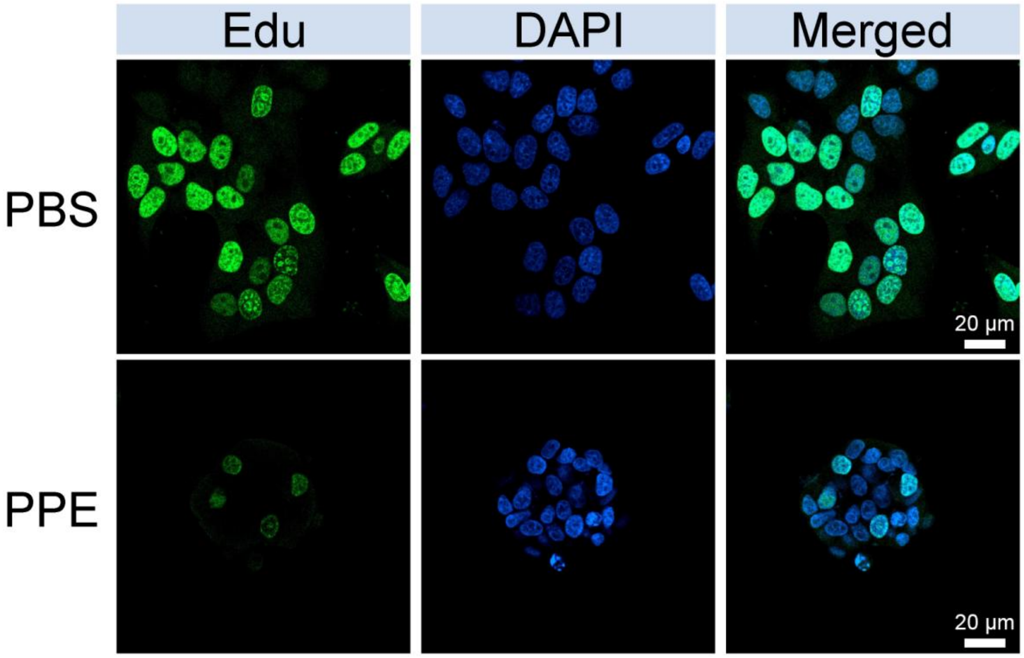

Figure S4. The cell proliferation of 3B cells after treatment with PBS or PPE (40 μg/mL) was evaluated by Edu assay. Green fluorescence from Edu-Alexa Fluor 488; blue fluorescence showed nucleus stained with DAPI. The scale bar is 20 μm.

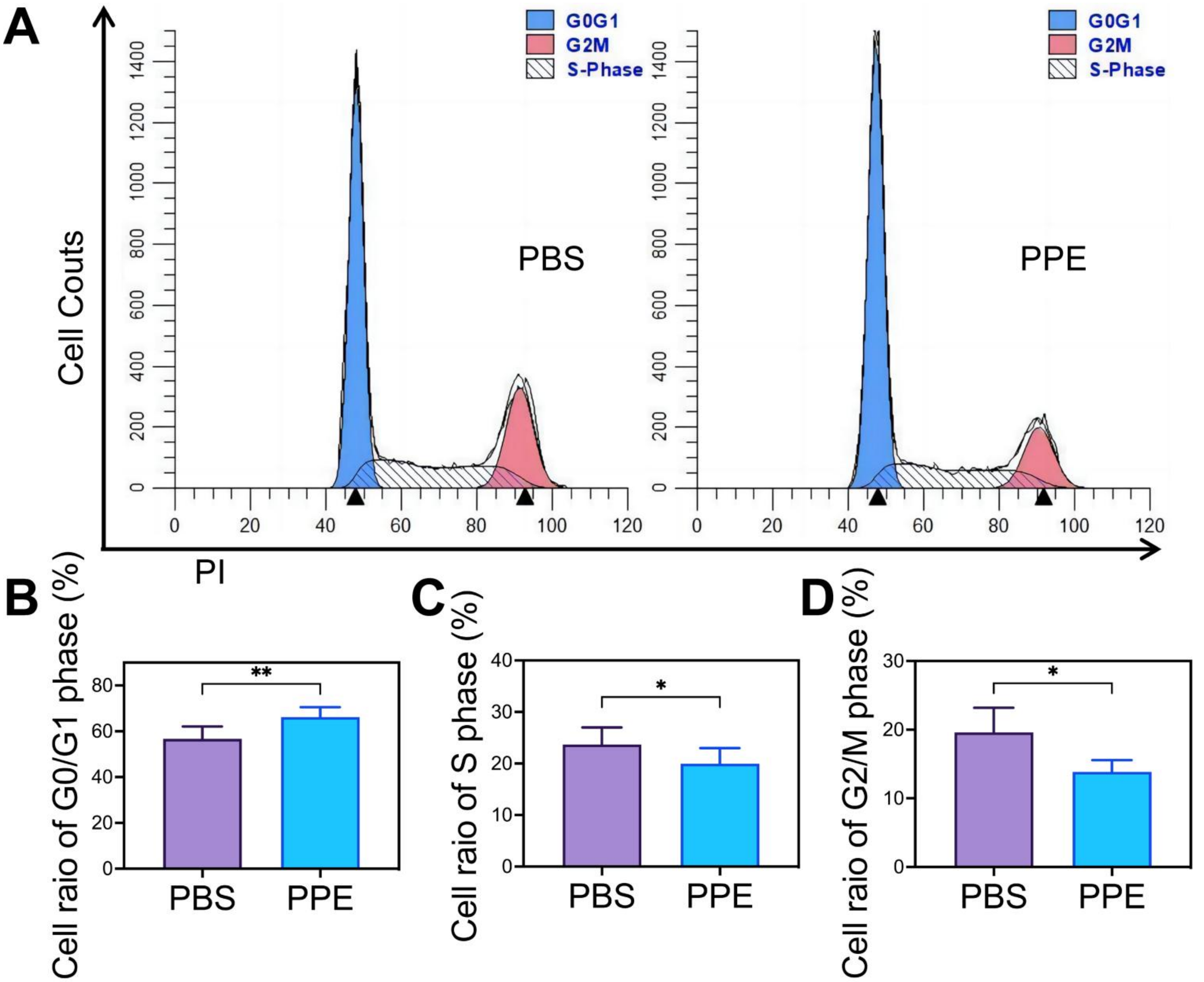

Figure S5. A, B, C, D) Cell cycle analysis of 3B cells after treatment with PBS or PPE was quantified by flow cytometry. The data are expressed as mean ± SD. $*p < 0.05$, $**p < 0.01$, $***p < 0.001$, $****p < 0.0001$.

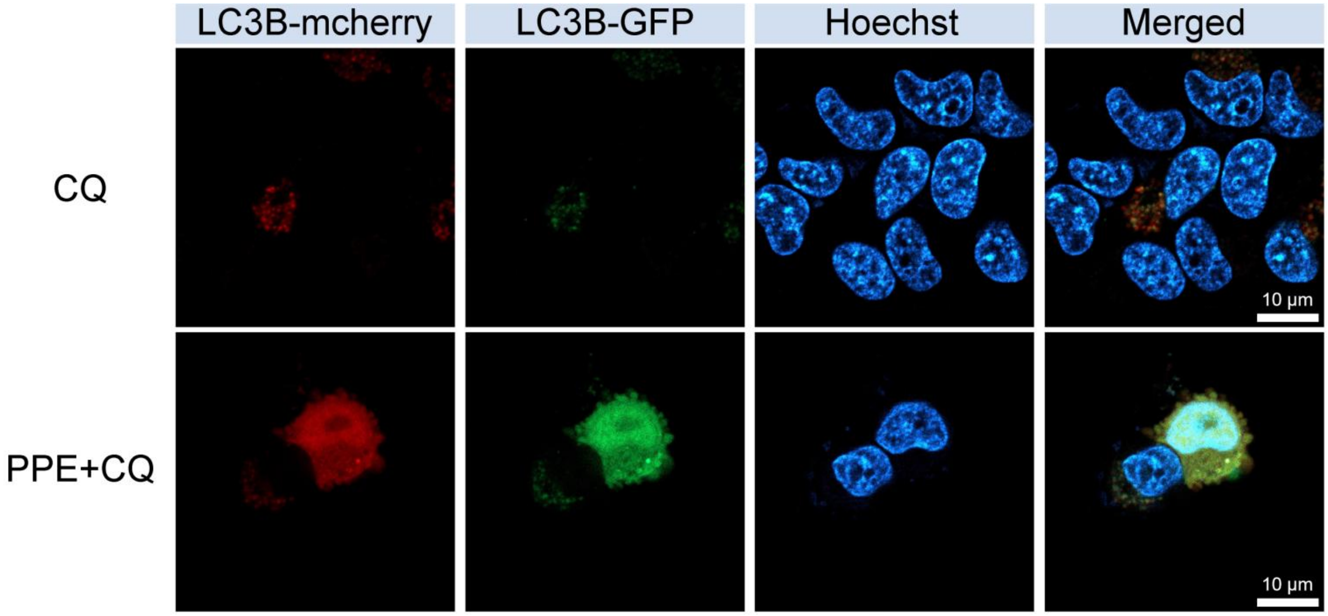

Figure S6. Fluorescence images of LC3B expression in 3B cells after different treatments. Red fluorescence from LC3B labeled with mcherry; green fluorescence from LC3B labeled with GFP; blue fluorescence showed nucleus stained with Hoechst33342. The scale bar is 10 μm.

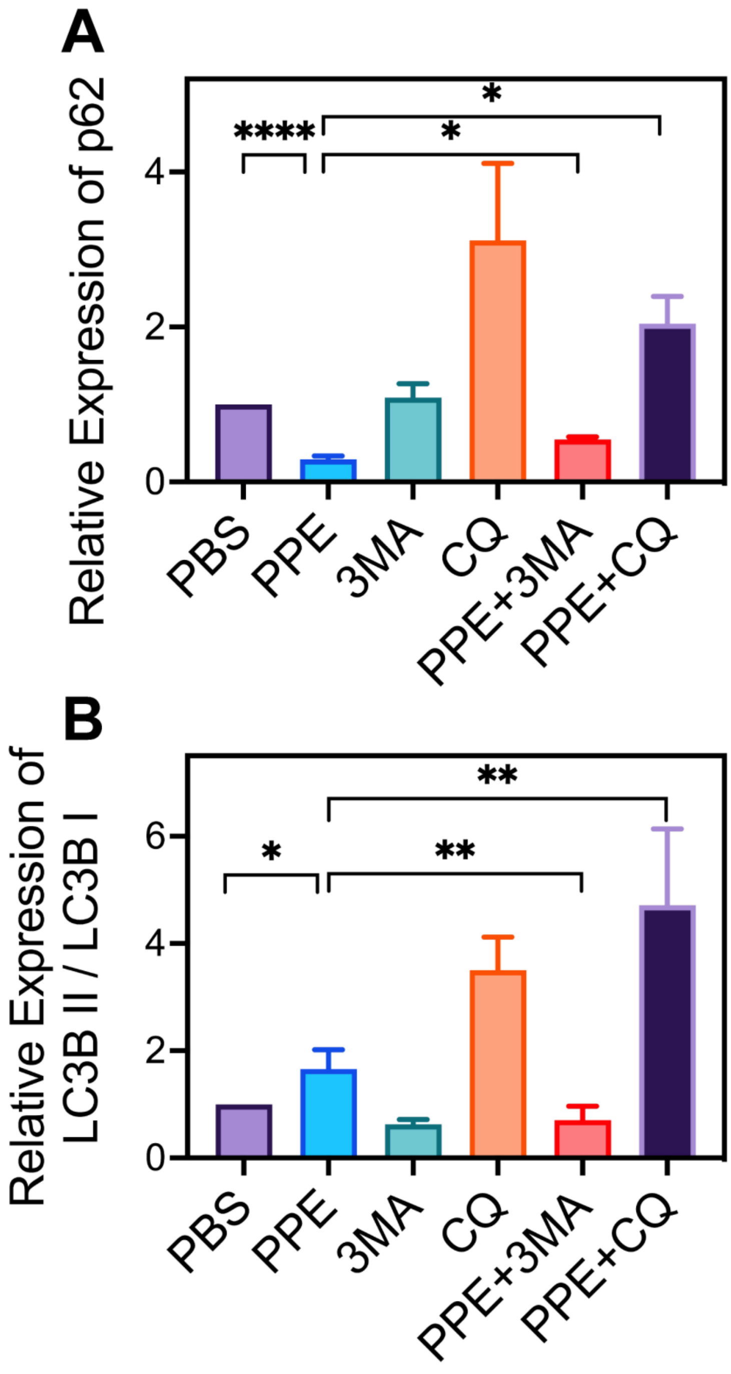

Figure S7. The expression of **A)** p62 and **B)** LC3B II/LC3B I in 3B cells after different treatments was quantified by immunoblot analysis. The data are expressed as mean ± SD. *$p < 0.05$, **$p < 0.01$, ***$p < 0.001$, ****$p < 0.0001$.

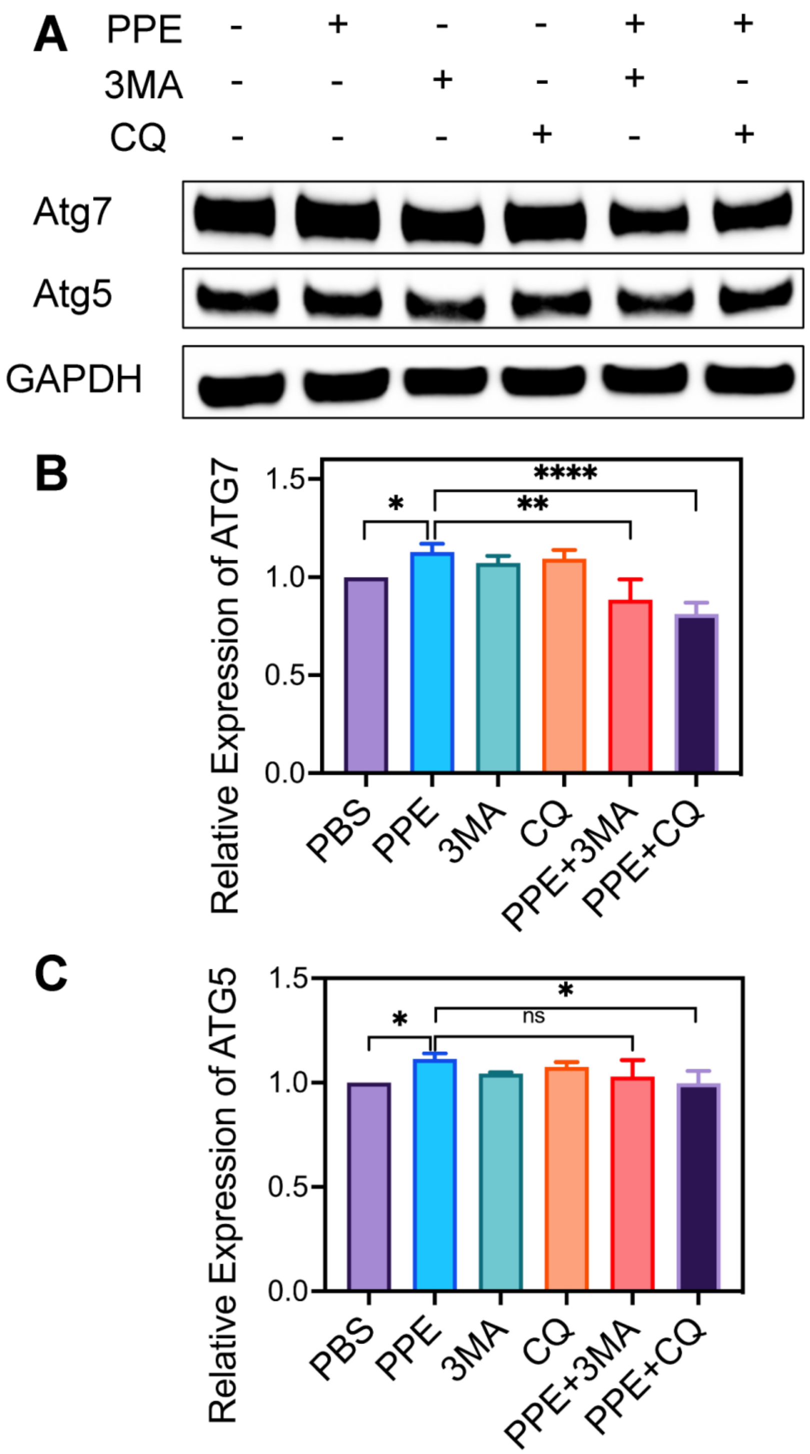

Figure S8. The expression of **A, B)** ATG7 and **A, C)** ATG5 in 3B cells after different treatments was quantified by immunoblot analysis. The data are expressed as mean ± SD. *$p < 0.05$, **$p < 0.01$, ***$p < 0.001$, ****$p < 0.0001$, ns: no statistical difference.

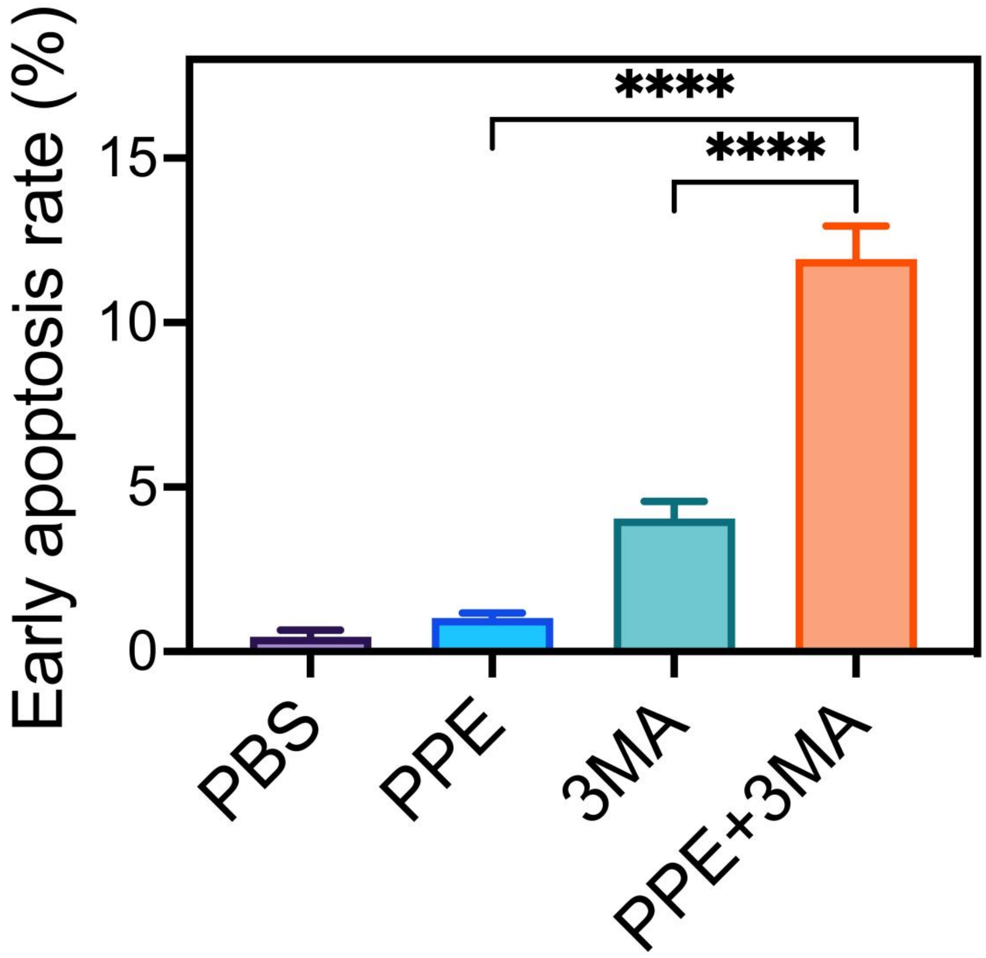

Figure S9. Early apoptosis of 3B cells after different treatments was quantified by flow cytometry. The data are expressed as mean ± SD. *$p < 0.05$, **$p < 0.01$, ***$p < 0.001$, ****$p < 0.0001$.

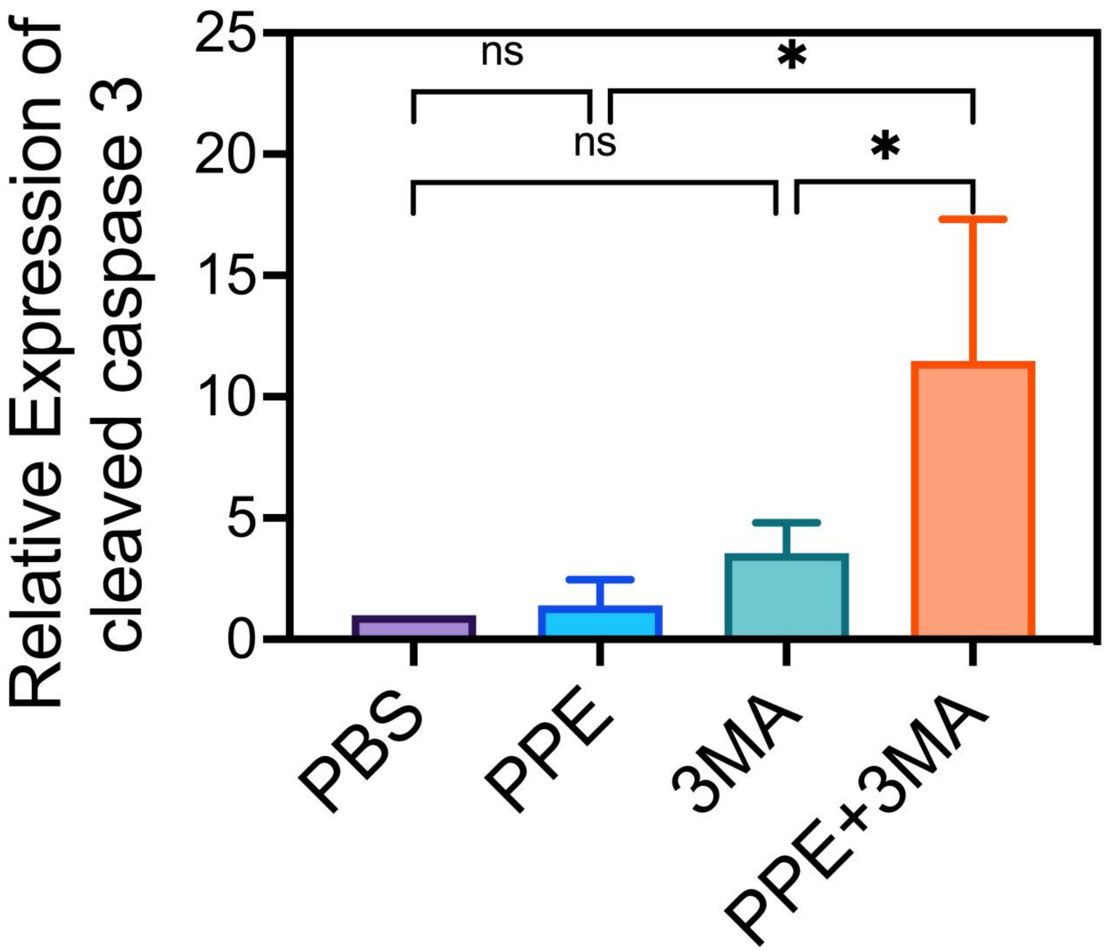

Figure S10. The expression of cleaved caspase 3 in 3B cells after different treatments was quantified by immunoblot analysis. The data are expressed as mean ± SD. *$p < 0.05$, **$p < 0.01$, ***$p < 0.001$, ****$p < 0.0001$, ns: no statistical difference.

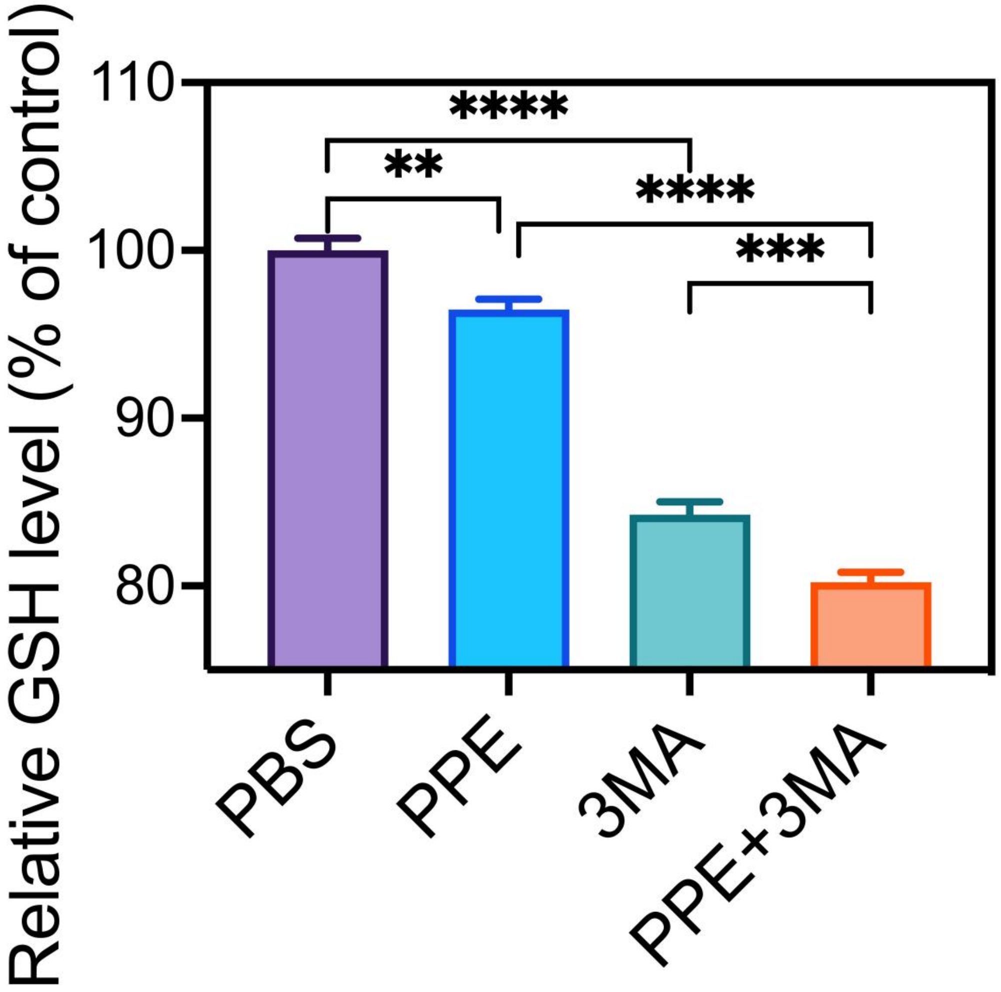

Figure S11. Evaluation of relative GSH levels in 3B cells after different treatments. The data are expressed as mean ± SD. *p < 0.05, **p < 0.01, ***p < 0.001, ****p < *0.0001*.

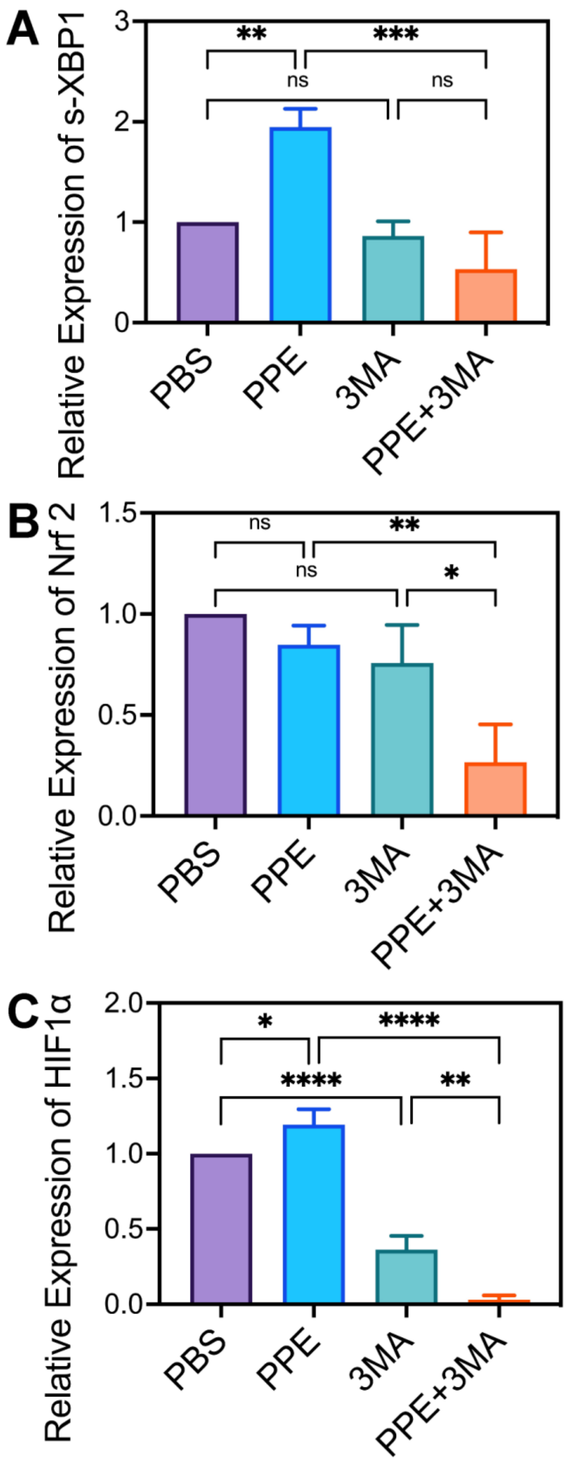

Figure S12. The expression of **A)** s-XBP1, **B)** Nrf2 and **C)** HIF1α in 3B cells after different treatments was quantified by immunoblot analysis. The data are expressed as mean ± SD. **p* < 0.05, ***p* < 0.01, ****p* < 0.001, *****p* < 0.0001, ns: no statistical difference.

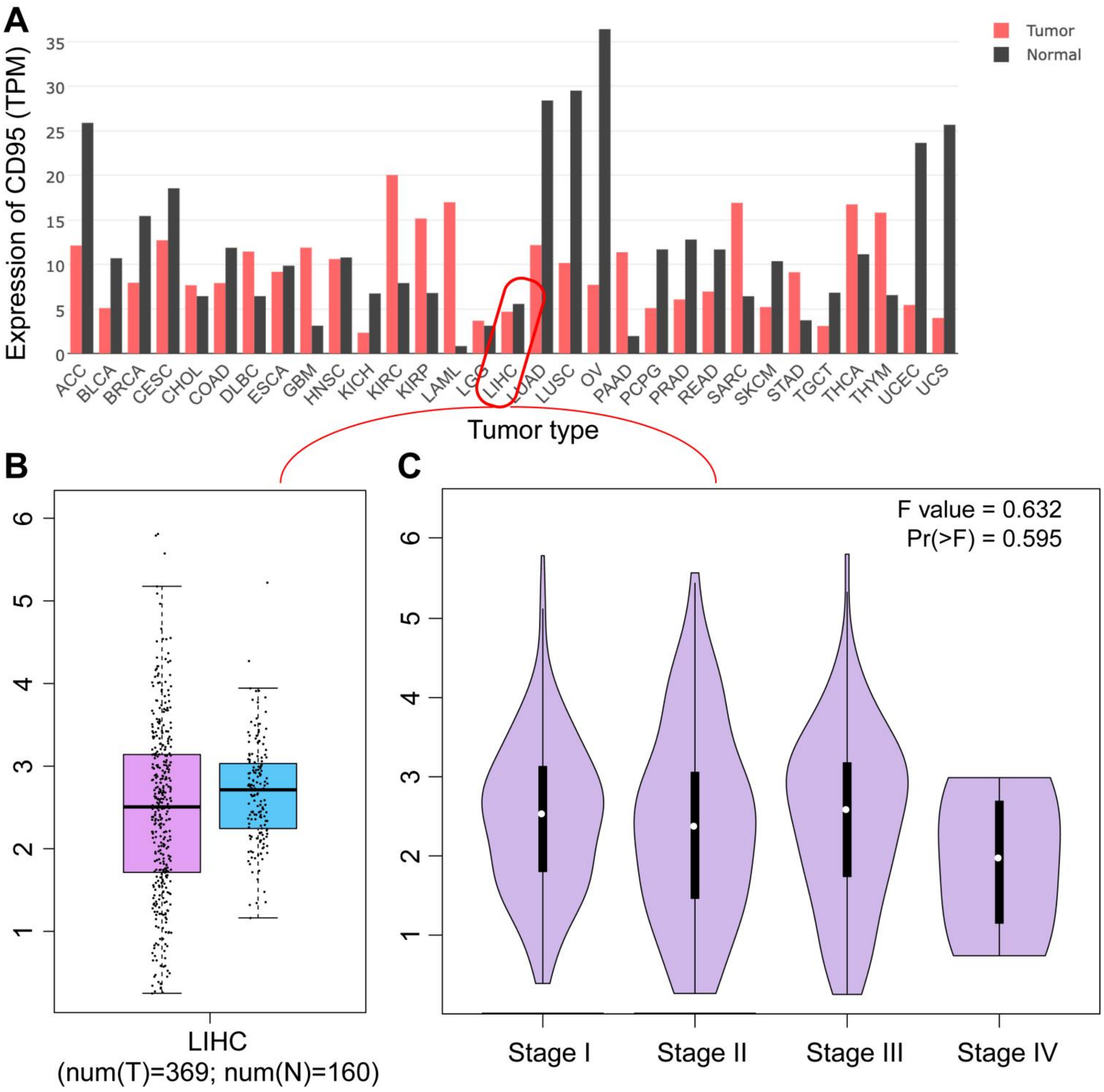

Figure S13. A) The expression of CD95 in different tumors compared with normal tissues. LIHC: liver hepatocellular carcinoma. **B)** The expression of CD95 in liver hepatocellular carcinoma compared with normal tissues. **C)** The expression of CD95 in different stages of liver hepatocellular carcinoma, created by GEPIA.

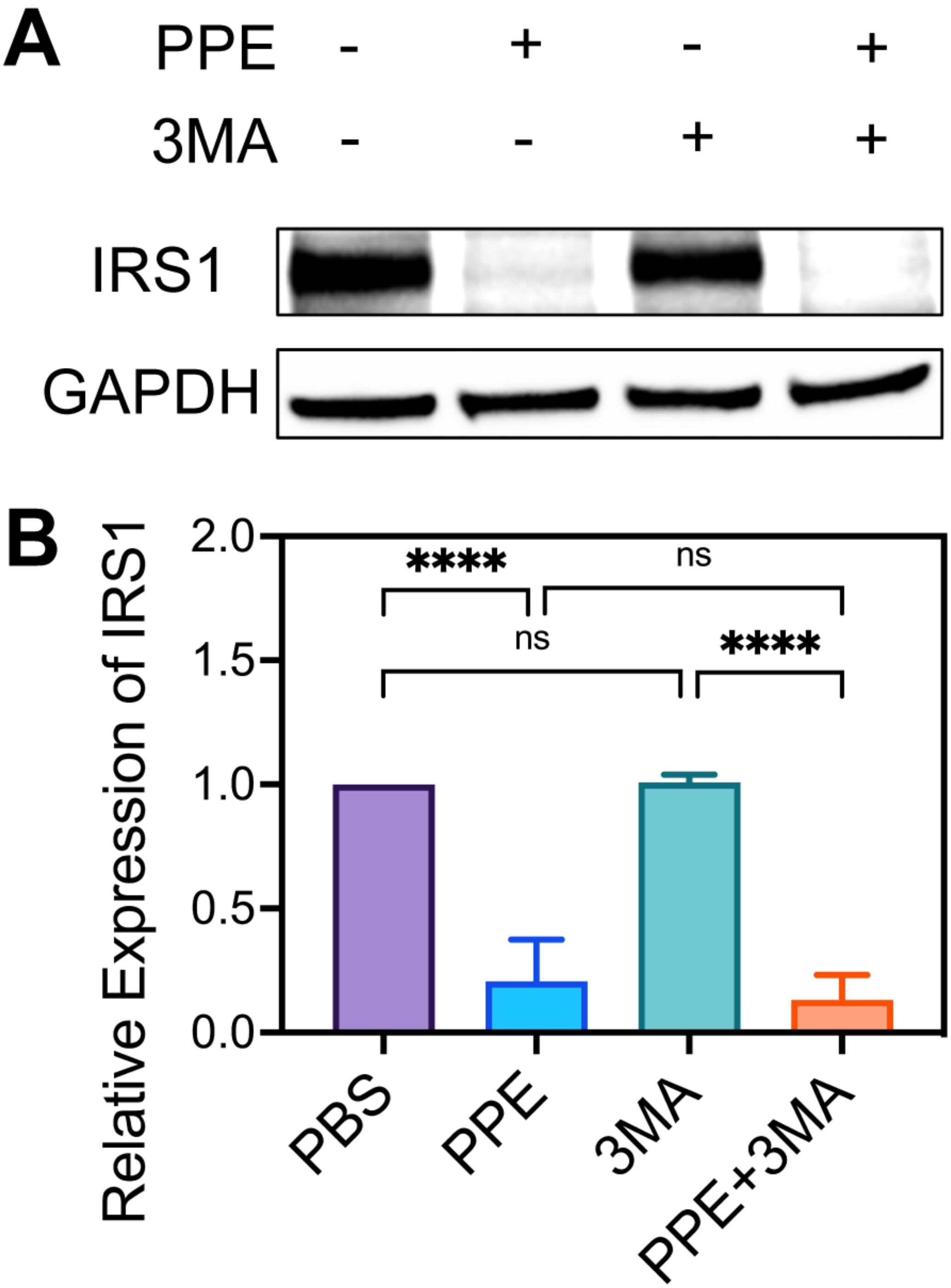

Figure S14. A, B) The expression of IRS1 in 3B cells after different treatments was quantified by immunoblot analysis. The data are expressed as mean ± SD. *$p < 0.05$, **$p < 0.01$, ***$p < 0.001$, ****$p < 0.0001$, ns: no statistical difference.

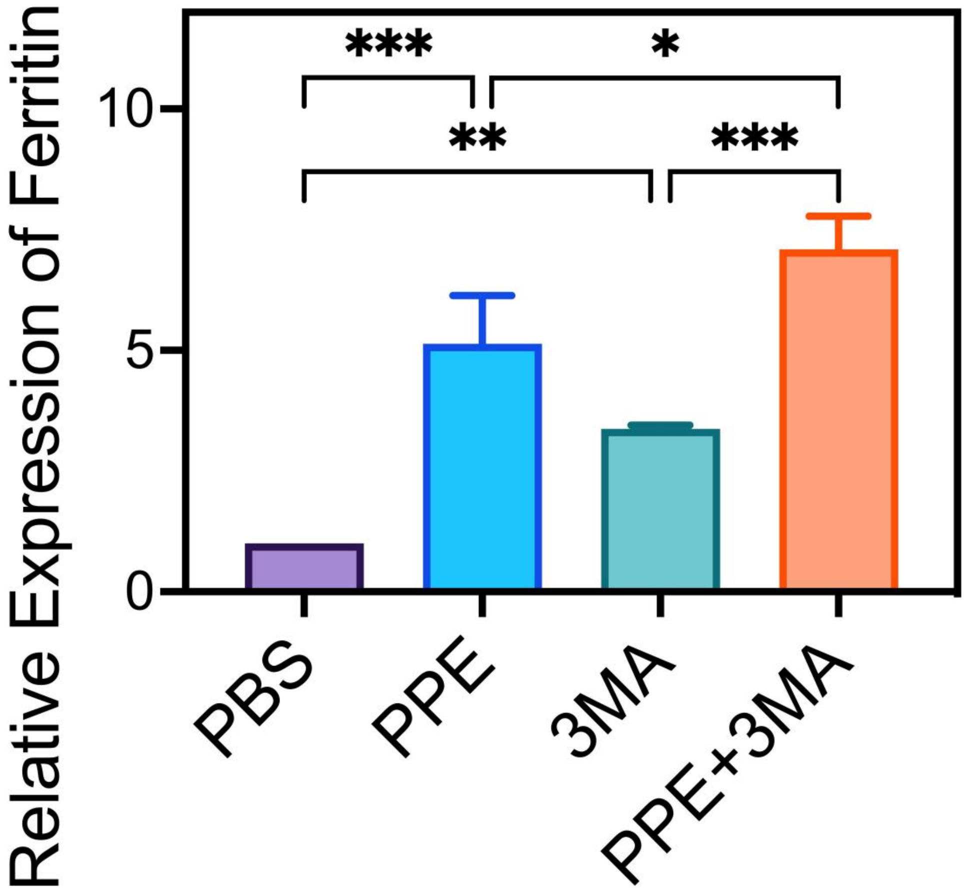

Figure S15. The expression of ferritin in 3B cells after different treatments was quantified by immunoblot analysis. The data are expressed as mean ± SD. $*p < 0.05$, $**p < 0.01$, $***p < 0.001$, $****p < 0.0001$.

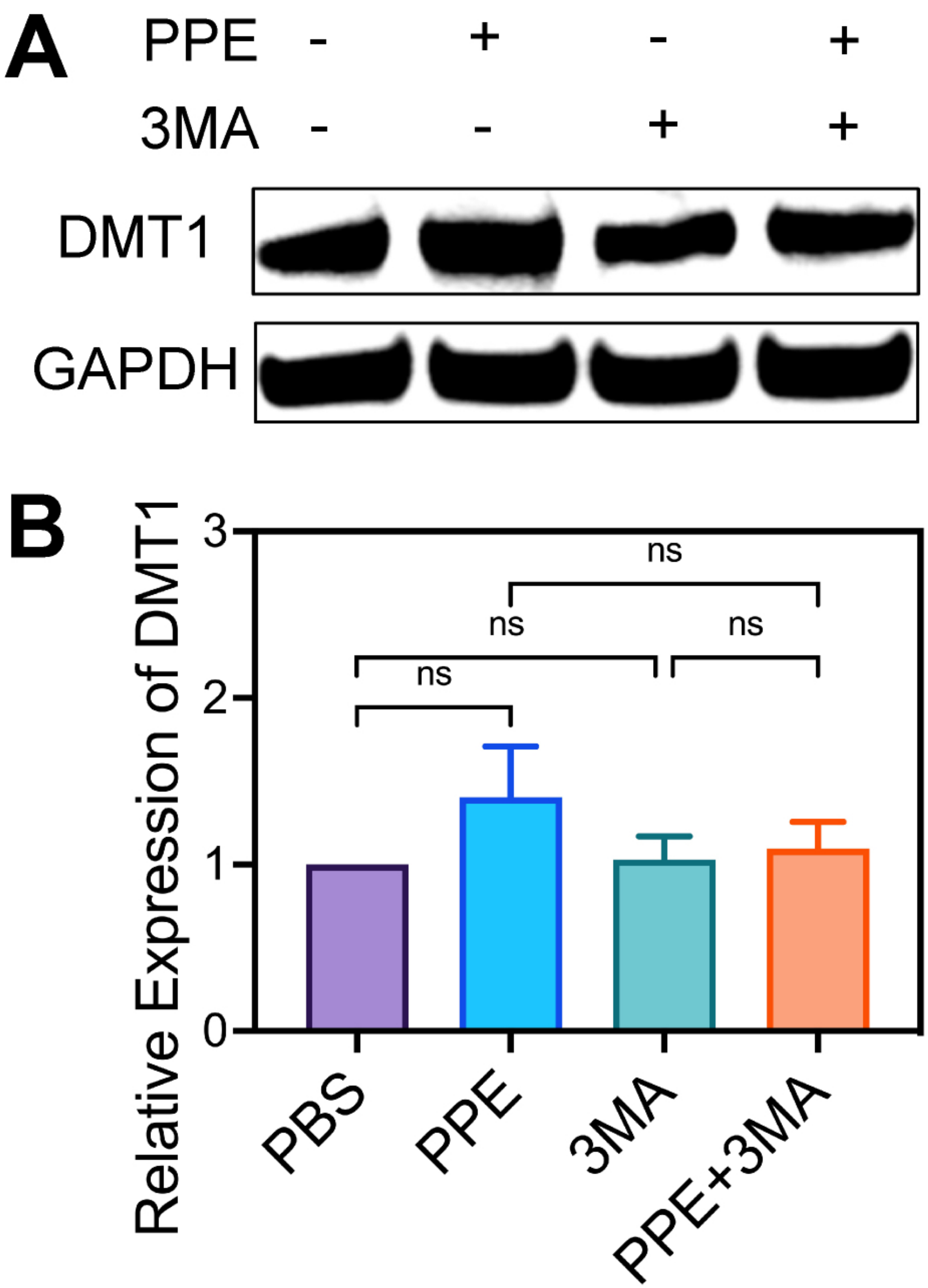

Figure S16. A, B) The expression of DMT1 in 3B cells after different treatments was quantified by immunoblot analysis. The data are expressed as mean ± SD. **p* < 0.05, ***p* < 0.01, ****p* < 0.001, *****p* < 0.0001, ns: no statistical difference.

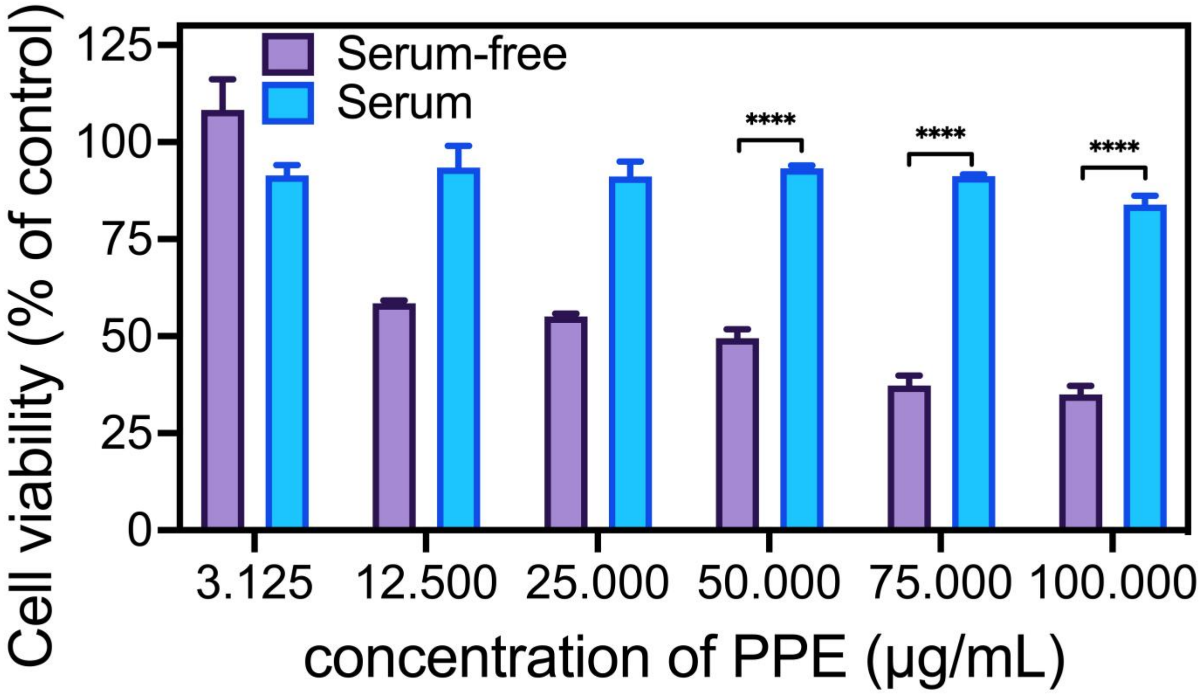

Figure S17. The cell viability of 3B cells after treatment with different concentrations of PPE in the serum or serum-free conditions was quantified by CCK8. The data are expressed as mean ± SD. *p < 0.05, **p < 0.01, ***p < 0.001, ****p < 0.0001.

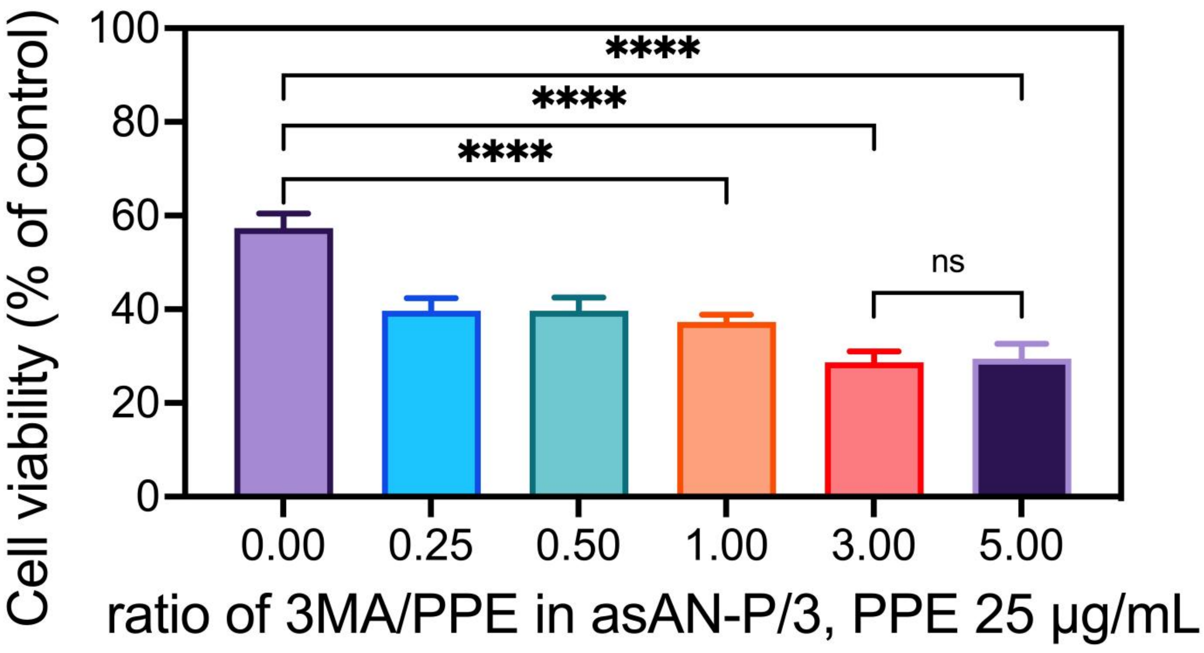

Figure S18. The cell viability of 3B cells after treatment with different ratios of 3MA/PPE in asAN-P/3 was quantified by CCK8. The data are expressed as mean ± SD. *p < 0.05, **p < 0.01, ***p < 0.001, ****p < 0.0001, ns: no statistical difference.

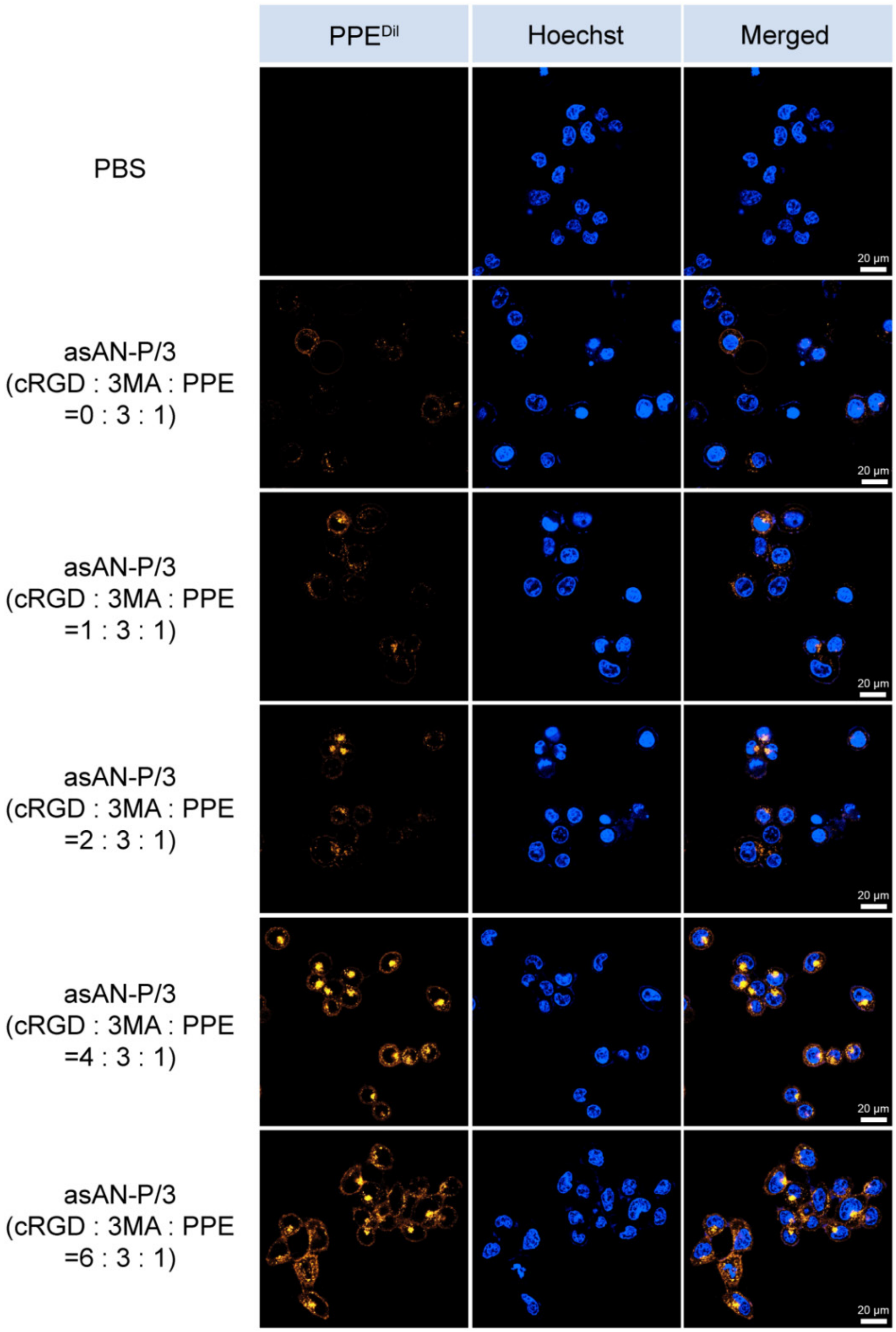

Figure S19. Confocal laser scanning microscopy (CLSM) images of 3B cells incubated with PBS or treatments with different ratios of cRGD/3MA in asAN-P/3 for 1 h. Orange fluorescence from PPE labeled with Dil; blue fluorescence indicates nuclei stained with Hoechst 33342. asAN-P/3: autophagy inhibitor-sensitized artificially activated neutrophils. The scale bar is 20 μm.

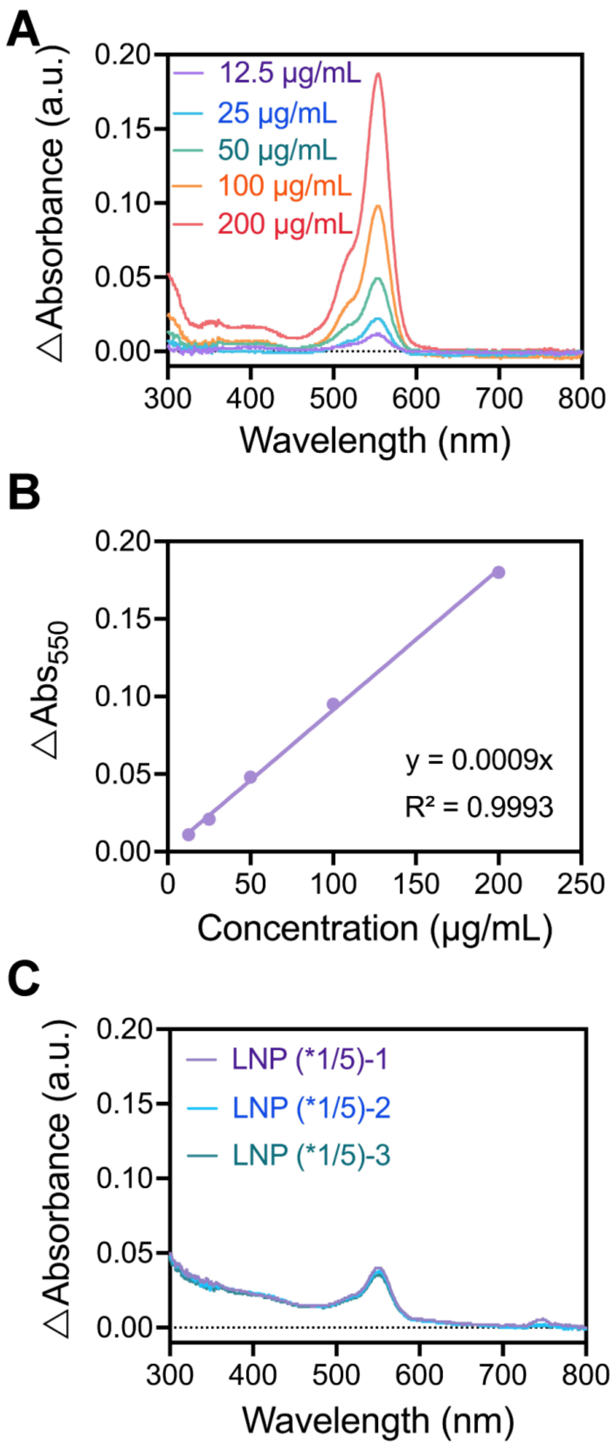

Figure S20. A) The ultraviolet-visible light spectrum of different concentrations of C16-cRGD^{RB}. **B)** The standard curve of C16-cRGD^{RB}. **C)** The ultraviolet-visible light spectrum of liposomes (0.3 mg/mL) prepared by C16-cRGD^{RB}.

Table S1. Assessment progress of cRGD density in liposomes

	LNP (*1/5)-1	LNP (*1/5)-2	LNP (*1/5)-3
△Abs_{550} (a.u.)	0.040	0.037	0.035
Concentration (μg/mL)	44.444	41.111	38.889
Initial Concentration (μg/mL)	222.222	205.556	194.444
Mass ratio	0.148	0.137	0.130
Relative density of cholesterol		1.067	
Relative density of Lecithin		1.031	
Relative density of Liposome (Estimation)		1.039	
Density of cRGD (g/cm^3)	0.154	0.142	0.135
Average Density of cRGD (g/cm^3)		0.144	

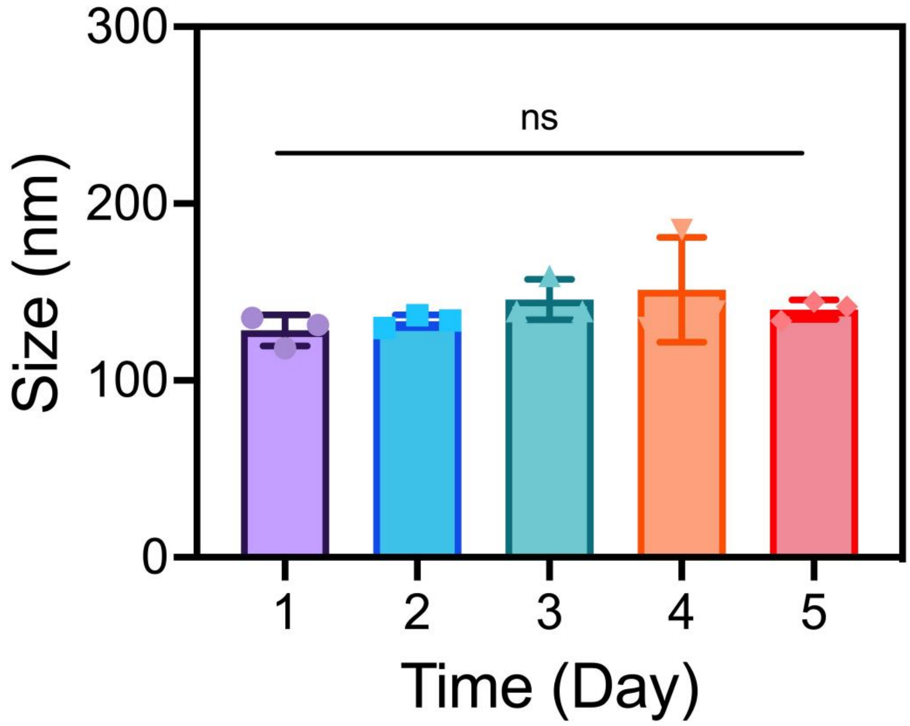

Figure S21. The hydrated diameters of asAN-P/3 in plasma on different days. asAN-P/3: autophagy inhibitor-sensitized artificially activated neutrophils. The data are expressed as mean ± SD. ns: no statistical difference.

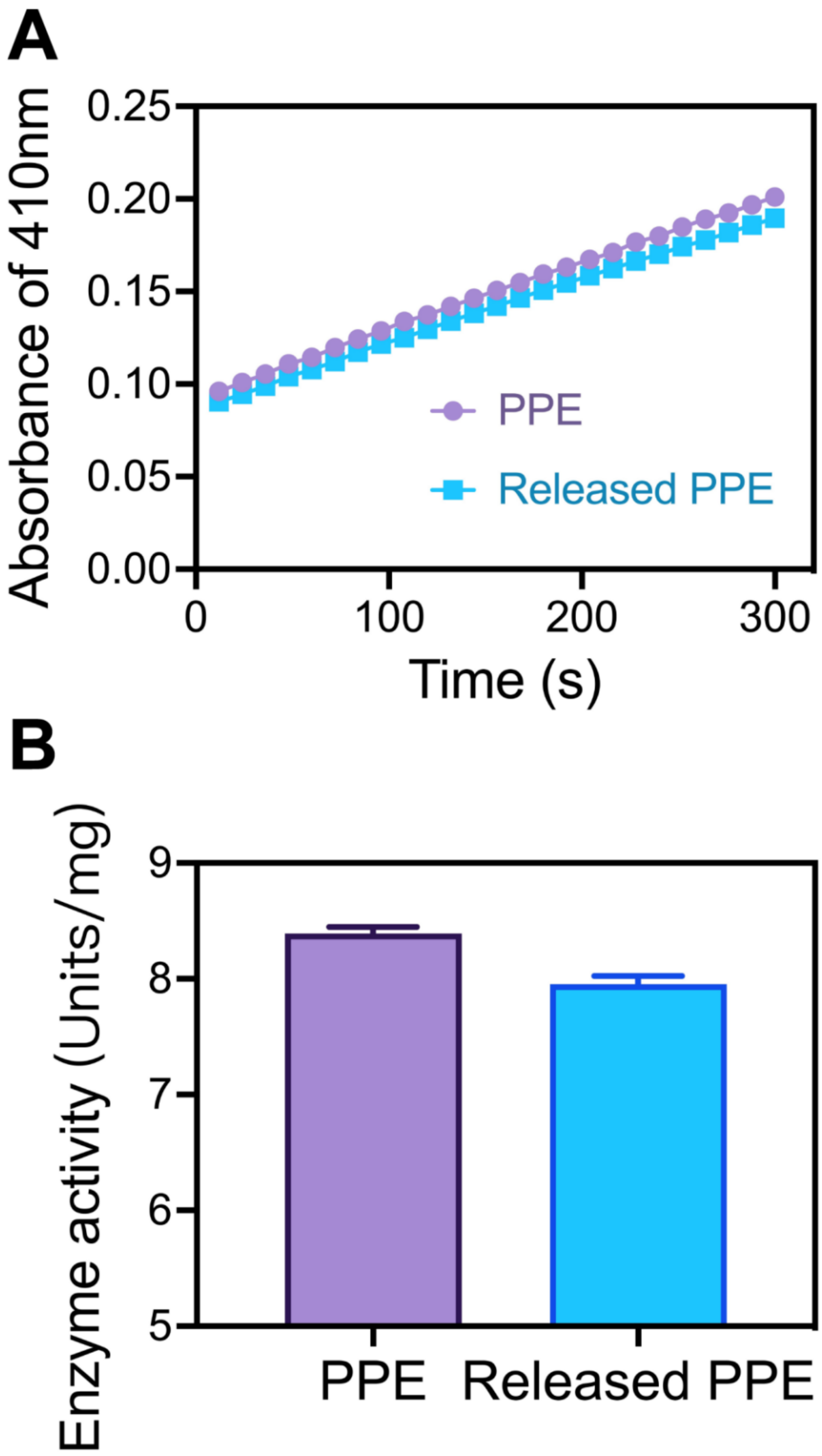

Figure S22. A, B) The enzyme activity of the free PPE and released PPE from asAN-P/3 was quantified by absorbance at 410nm. asAN-P/3: autophagy inhibitor-sensitized artificially activated neutrophils. The data are expressed as mean ± SD.

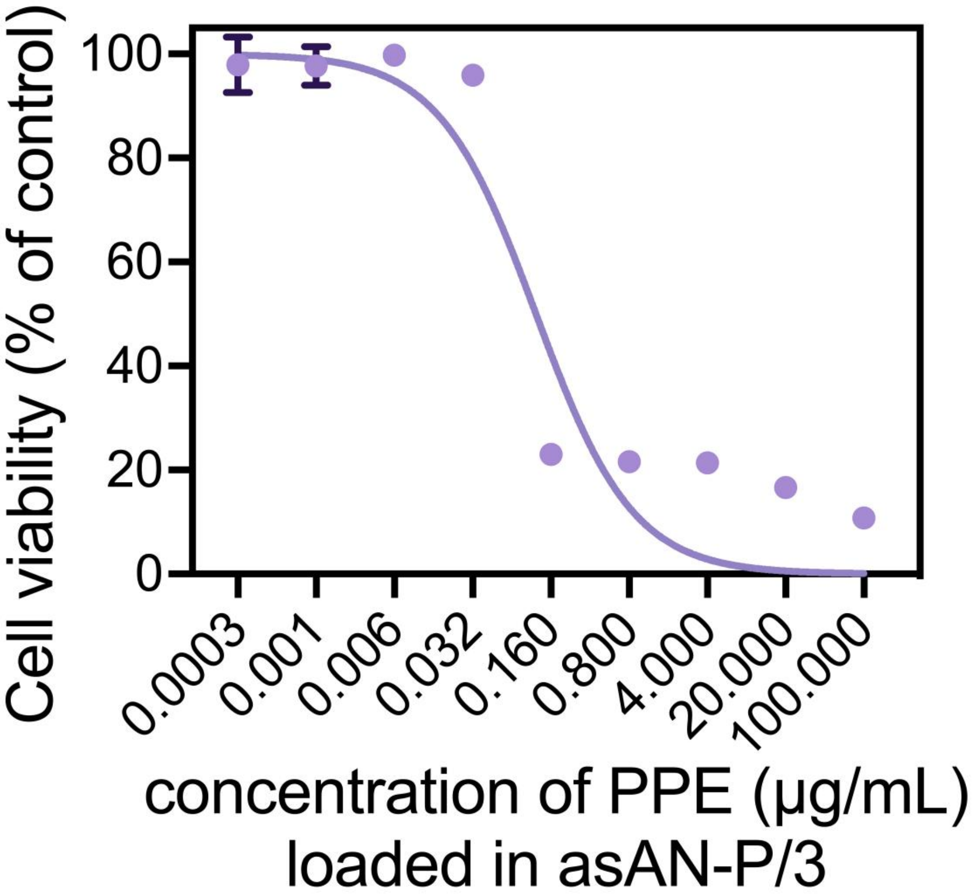

Figure S23. Concentration-dependent cytotoxicity evaluation of 3B cells after treatment with different concentrations of asAN-P/3. The concentrations of the nanomedicines were dependent on PPE, the concentration of 3MA was 3.67 times that of PPE. asAN-P/3: autophagy inhibitor-sensitized artificially activated neutrophils. The data are expressed as mean ± SD.

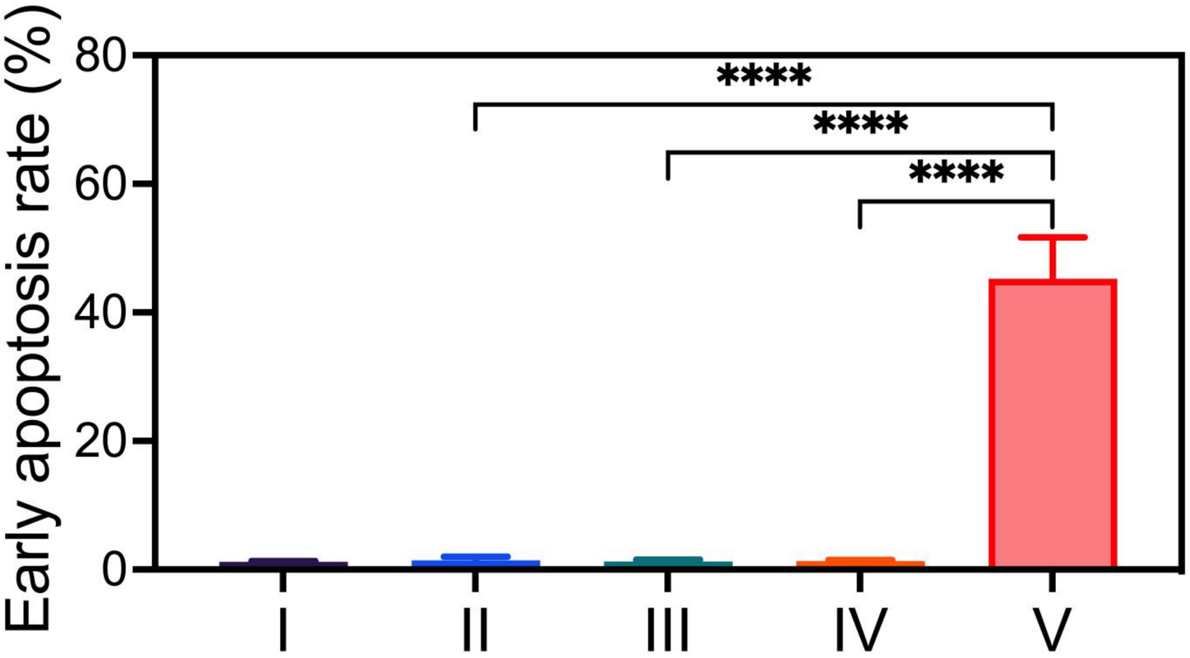

Figure S24. Early apoptosis of 3B cells after different treatments was quantified by flow cytometry. I: **PBS**; II: **PPE+3MA**; III: **cL-P;** IV: **cL-3;** V: **asAN-P/3.** cL-P: cRGD modified liposomes loaded with PPE; cL-3: cRGD modified liposomes loaded with 3MA; asAN-P/3: autophagy inhibitor-sensitized artificially activated neutrophils. The data are expressed as mean ± SD. *$p < 0.05$, **$p < 0.01$, ***$p < 0.001$, ****$p < 0.0001$.

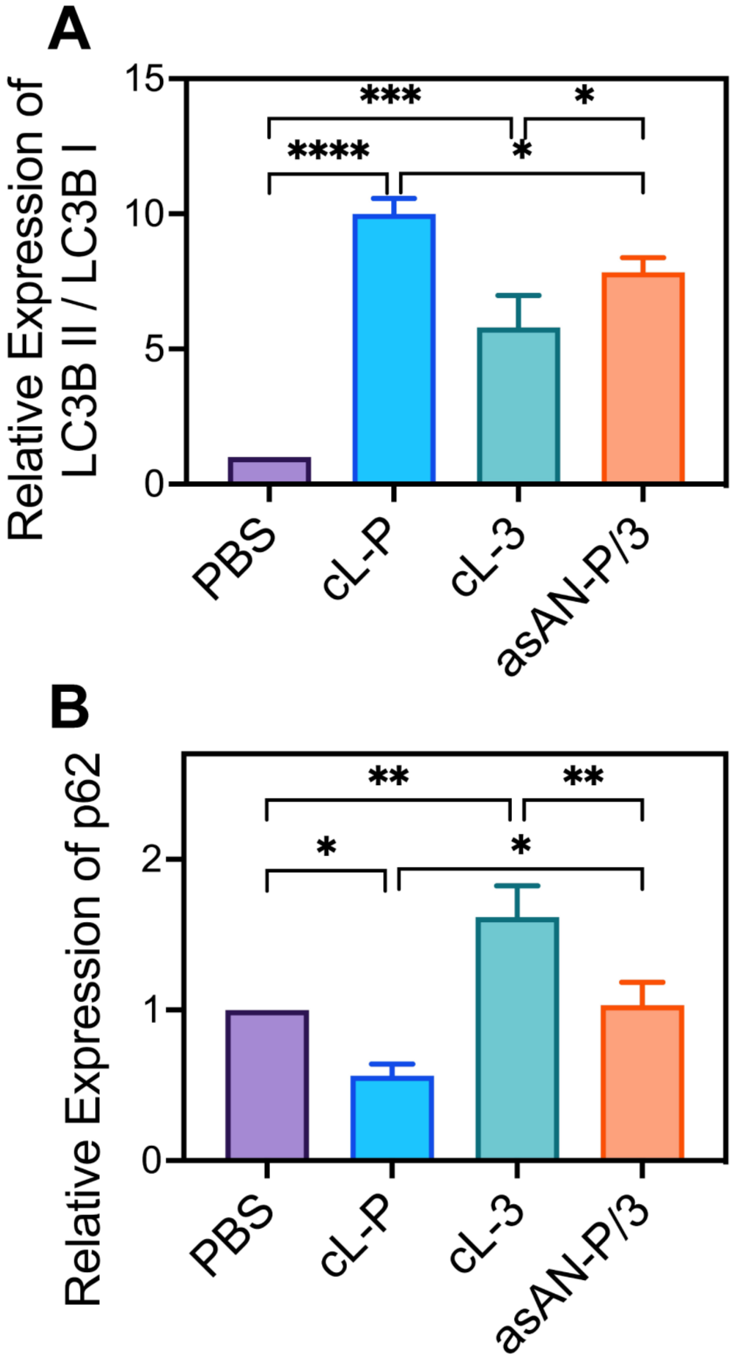

Figure S25. The expression of **A)** LC3B II/LC3B I and **B)** p62 in 3B cells after different treatments was quantified by immunoblot analysis. cL-P: cRGD modified liposomes loaded with PPE; cL-3: cRGD modified liposomes loaded with 3MA; asAN-P/3: autophagy inhibitor-sensitized artificially activated neutrophils. The data are expressed as mean ± SD. *$p < 0.05$, **$p < 0.01$, ***$p < 0.001$, ****$p < 0.0001$.

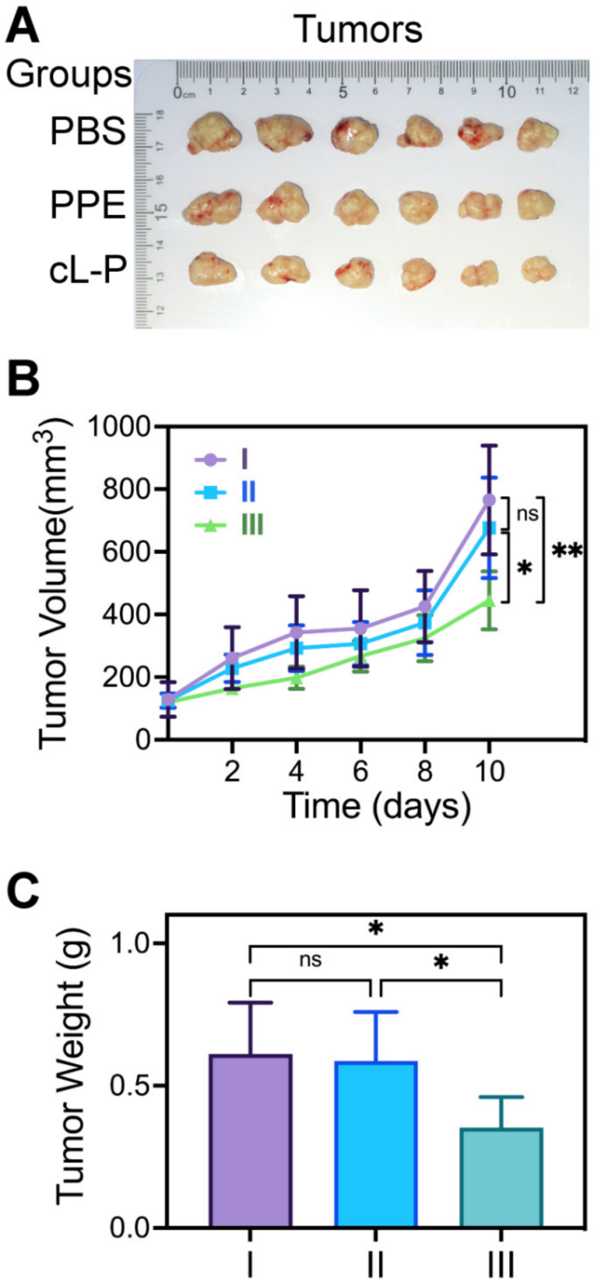

Figure S26. A) The photograph of *ex vivo* tumors from mice after different treatments on day 10. **B)** The growth curves of tumors of the mice after different treatments. **C)** The tumor weights of *ex vivo* tumors from mice after different treatments on day 10. I: **PBS**; II: **PPE**; III: **cL-P.** cL-P: cRGD modified liposomes loaded with PPE. The data are expressed as mean ± SD. *$p < 0.05$, **$p < 0.01$, ***$p < 0.001$, ****$p < 0.0001$, ns: no statistical difference.

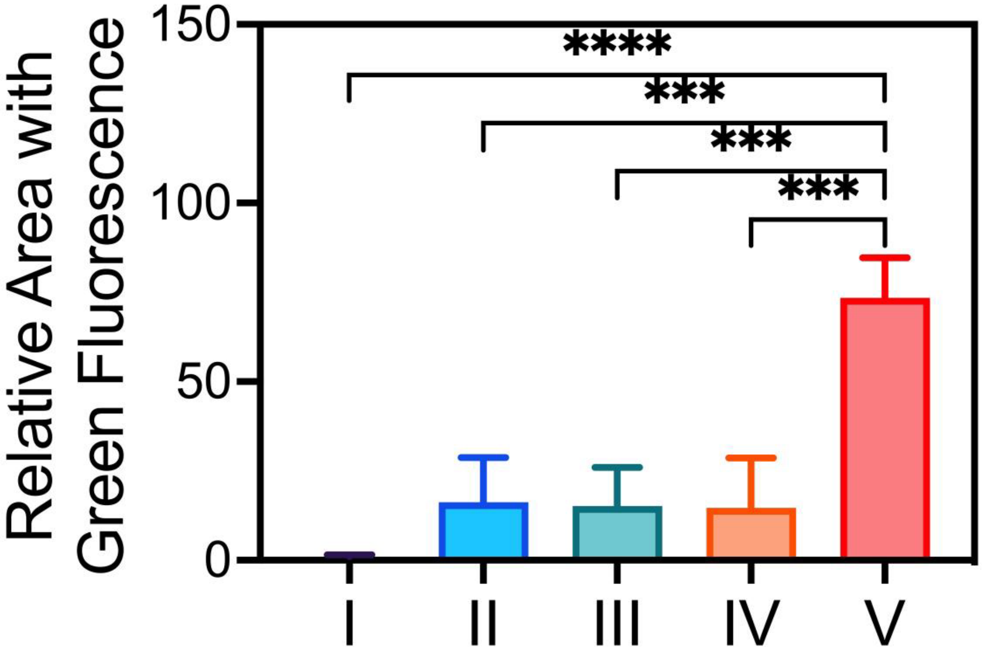

Figure S27. The quantification of the relative area with green fluorescence by TUNEL assay. I: **PBS**; II: **PPE+3MA**; III: **cL-P;** IV: **cL-3;** V: **asAN-P/3.** cL-P: cRGD modified liposomes loaded with PPE; cL-3: cRGD modified liposomes loaded with 3MA; asAN-P/3: autophagy inhibitor-sensitized artificially activated neutrophils. The data are expressed as mean ± SD. $*p < 0.05$, $**p < 0.01$, $***p < 0.001$, $****p < 0.0001$.

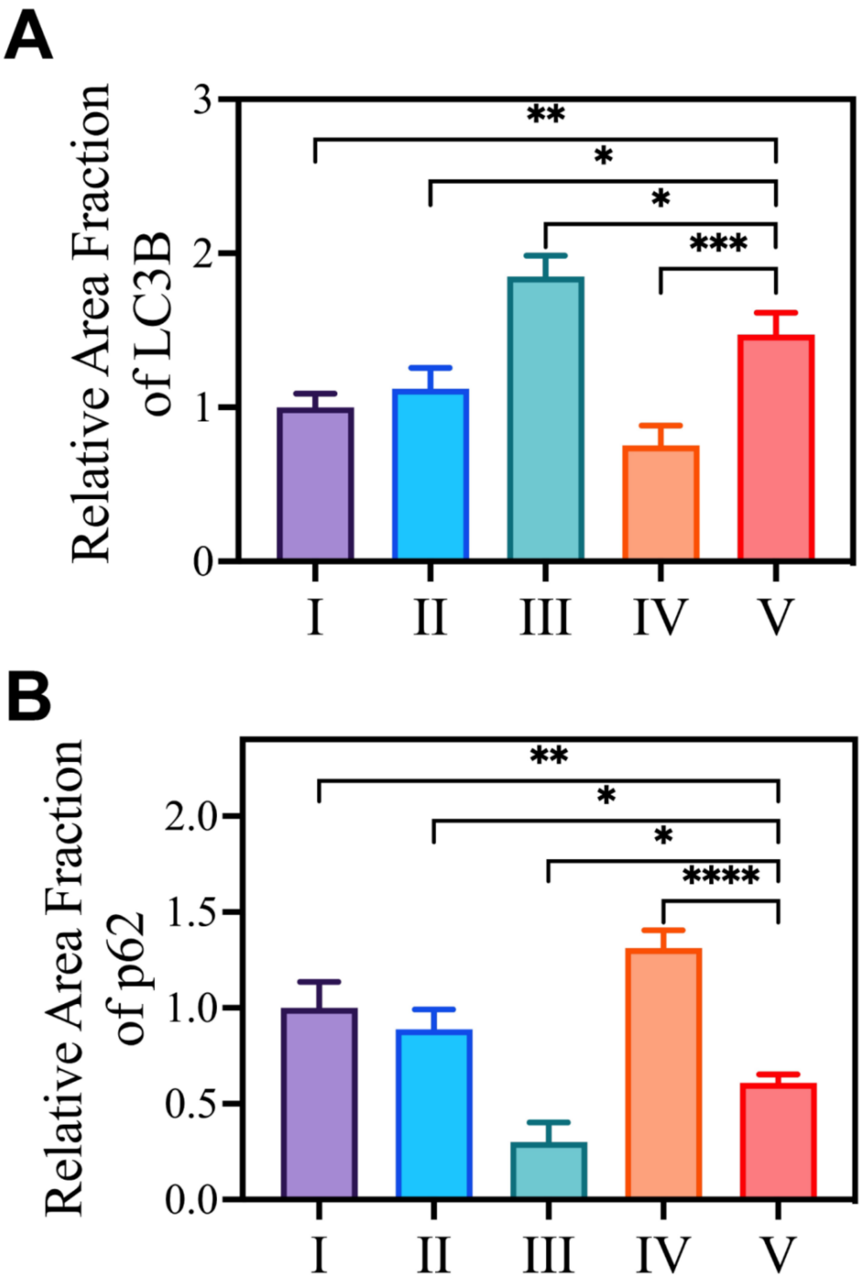

Figure S28. The quantification of **A)** LC3B and **B)** p62 immunohistochemistry assays of tumors was from mice after different treatments on day 10. I: **PBS**; II: **PPE+3MA**; III: **cL-P;** IV: **cL-3;** V: **asAN-P/3.** cL-P: cRGD modified liposomes loaded with PPE; cL-3: cRGD modified liposomes loaded with 3MA; asAN-P/3: autophagy inhibitor-sensitized artificially activated neutrophils. The data are expressed as mean ± SD. *$p < 0.05$, **$p < 0.01$, ***$p < 0.001$, ****$p < 0.0001$.

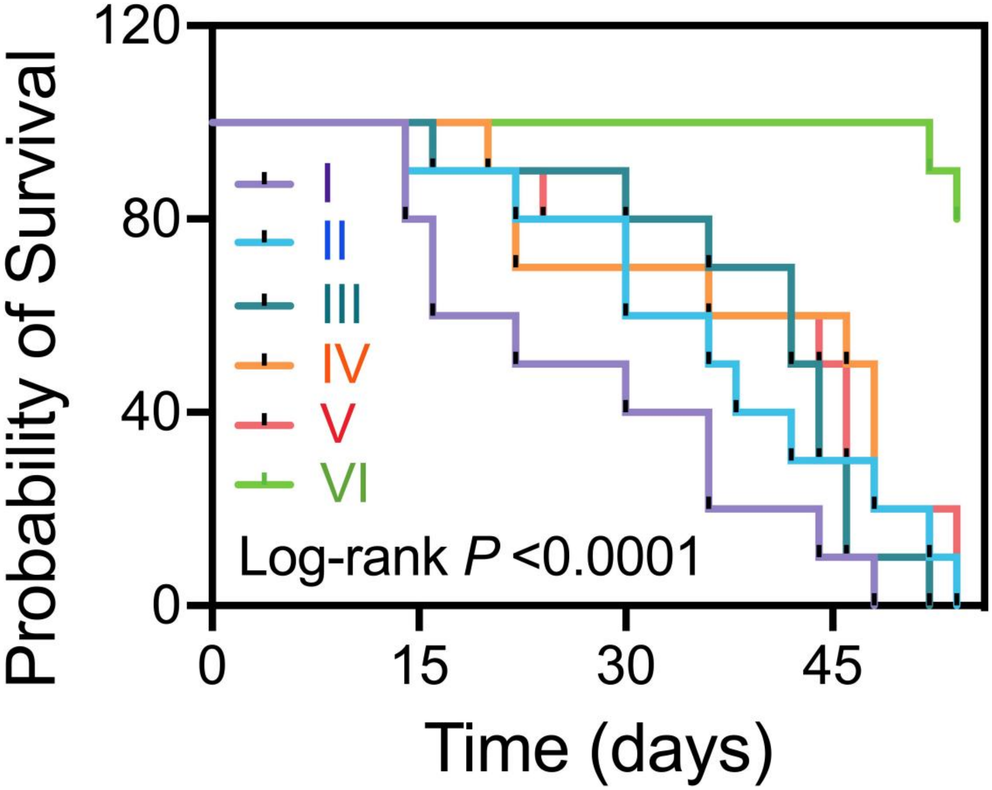

Figure S29. The survival curves of the mice after different treatments. I: **PBS**; II: **PPE**; III: **PPE+3MA**; IV: **cL-P;** V: **cL-3;** VI: **asAN-P/3.** cL-P: cRGD modified liposomes loaded with PPE; cL-3: cRGD modified liposomes loaded with 3MA; asAN-P/3: autophagy inhibitor-sensitized artificially activated neutrophils.

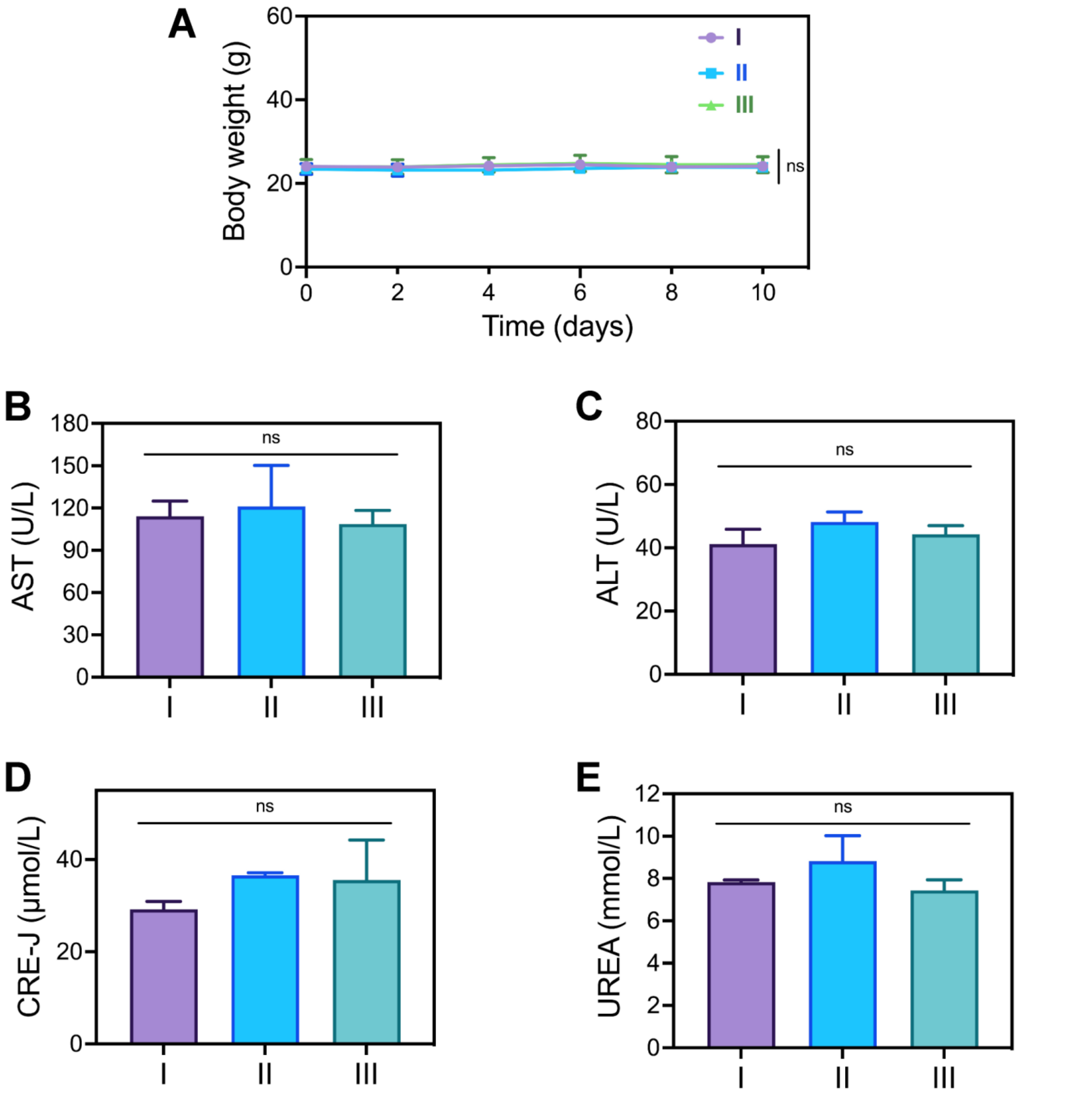

Figure S30. A) The curves of the body weights of the mice after different treatments at different time points. The hepatic function and renal function were evaluated by the quantification of **B)** glutamic oxaloacetic transaminase (AST), **C)** glutamic pyruvic transaminase (ALT), **D)** creatinine (CRE-J) and **E)** urea from mice after different treatments on day 10. I: **PBS**; II: **PPE**; III: **cL-P**. cL-P: cRGD modified liposomes loaded with PPE. The data are expressed as mean ± SD. ns: no statistical difference.

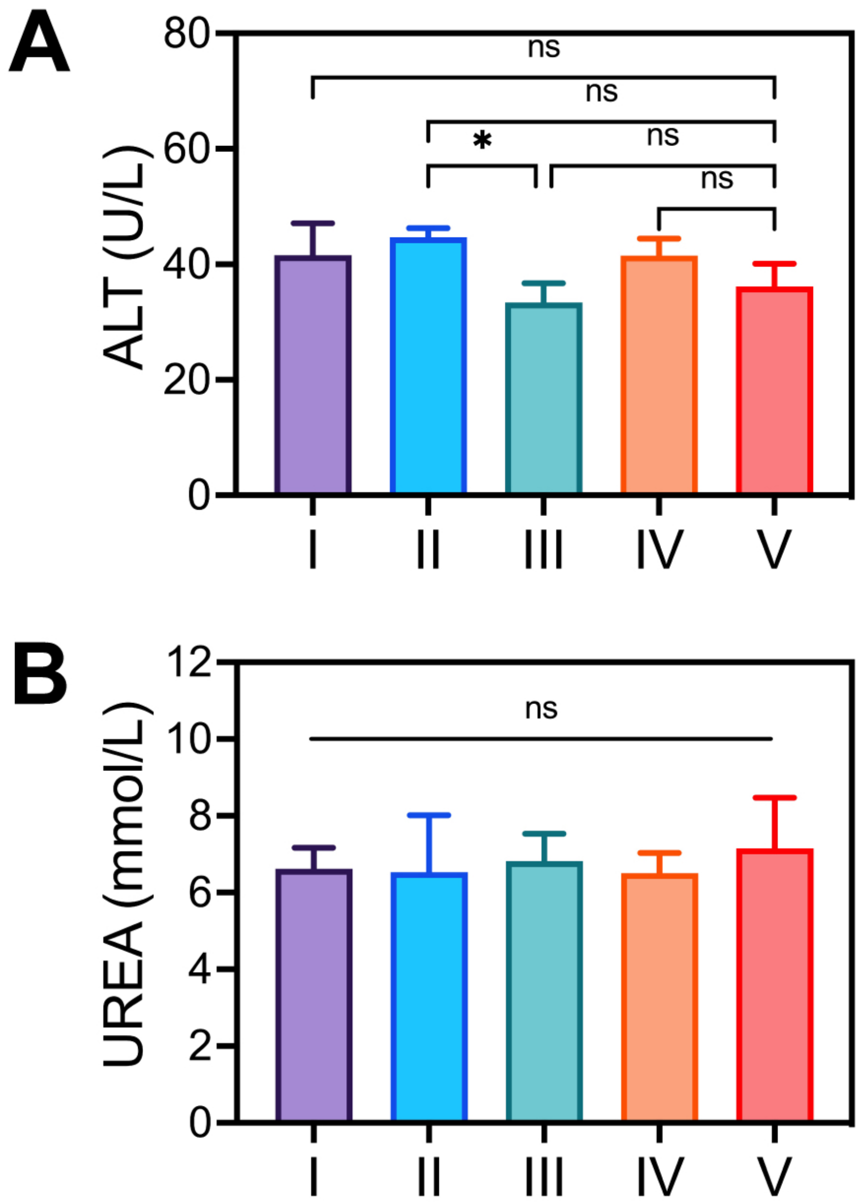

Figure S31. The hepatic function and renal function were evaluated by the quantification of **A)** glutamic pyruvic transaminase (ALT) and **B)** urea from mice after different treatments on day 10. I: **PBS**; II: **PPE+3MA**; III: **cL-P;** IV: **cL-3;** V: **asAN-P/3.** cL-P: cRGD modified liposomes loaded with PPE; cL-3: cRGD modified liposomes loaded with 3MA; asAN-P/3: autophagy inhibitor-sensitized artificially activated neutrophils. The data are expressed as mean ± SD. *$p < 0.05$, **$p < 0.01$, ***$p < 0.001$, ****$p < 0.0001$, ns: no statistical difference.

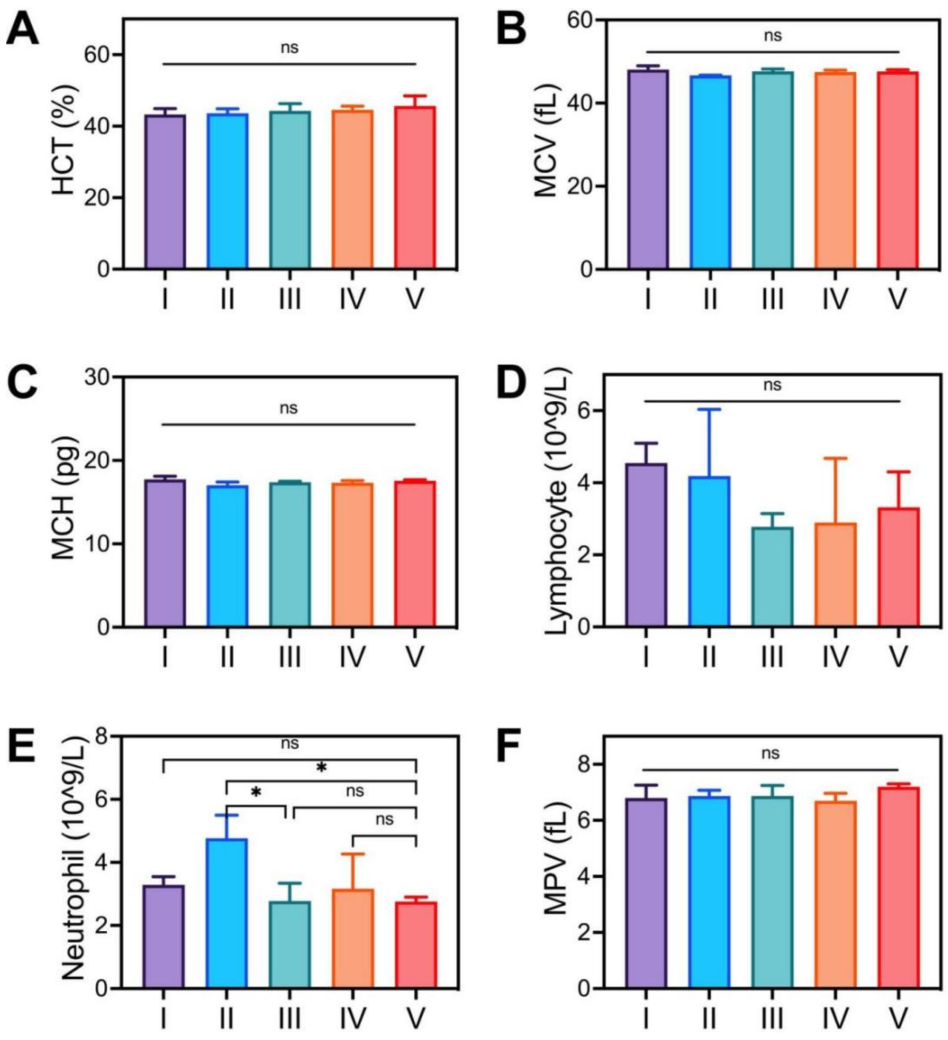

Figure S32. The hemolysis reaction and anemia evaluated by the quantification of **A)** hematocrit value (HCT), **B)** mean corpuscular volume (MCV) and **C)** mean corpuscular hemoglobin (MCH). The inflammatory state evaluated by the quantification of **D)** lymphocyte and **E)** neutrophil and the coagulation function evaluated by the quantification of **F)** mean platelet volume (MPV) were from mice after different treatments on day 10. I: **PBS**; II: **PPE+3MA**; III: **cL-P;** IV: **cL-3;** V: **asAN-P/3.** cL-P: cRGD modified liposomes loaded with PPE; cL-3: cRGD modified liposomes loaded with 3MA; asAN-P/3: autophagy inhibitor-sensitized artificially activated neutrophils. The data are expressed as mean ± SD. *$p < 0.05$, **$p < 0.01$, ***$p < 0.001$, ****$p < 0.0001$, ns: no statistical difference.

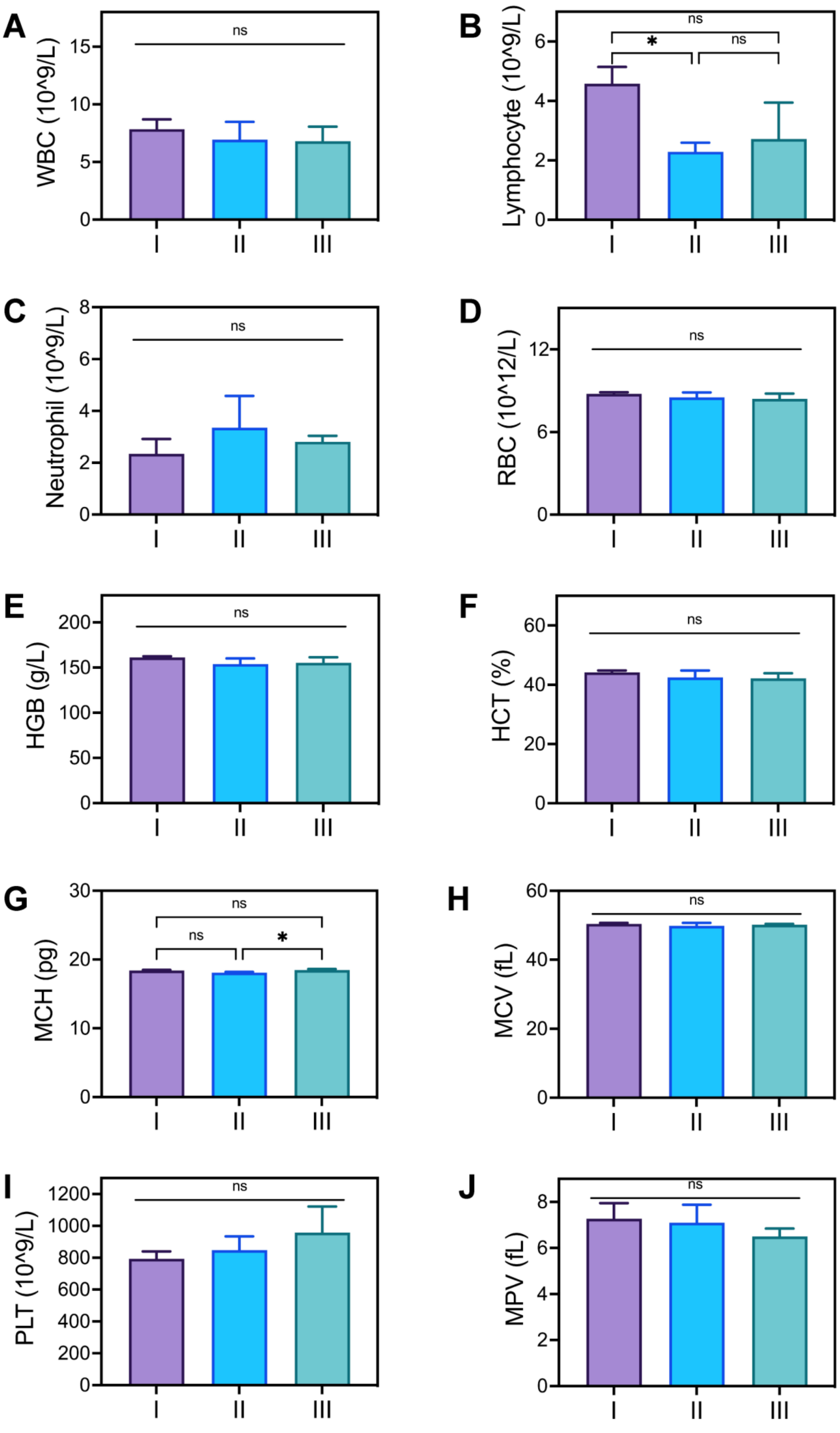
A
ns
WBC (10^9/L)
0
5
10
15
I
II
III
B
ns
*
ns
Lymphocyte (10^9/L)
0
2
4
6
I
II
III
C
ns
Neutrophil (10^9/L)
0
2
4
6
8
I
II
III
D
ns
RBC (10^12/L)
0
6
12
I
II
III
E
ns
HGB (g/L)
0
50
100
150
200
I
II
III
F
ns
HCT (%)
0
20
40
60
I
II
III
G
ns
ns
*
MCH (pg)
0
10
20
30
I
II
III
H
ns
MCV (fL)
0
20
40
60
I
II
III
I
ns
PLT (10^9/L)
0
200
400
600
800
1000
1200
I
II
III
J
ns
MPV (fL)
0
2
4
6
8
I
II
III

Figure S33. The inflammatory state evaluated by the quantification of **A)** white blood cells (WBC), **B)** lymphocyte and **C)** neutrophils, hemolysis reaction and anemia evaluated by the quantification of **D)** red blood cells (RBC), **E)** hemoglobin (HGB), **F)** hematocrit value (HCT), **G)** mean corpuscular hemoglobin (MCH) and **H)** mean corpuscular volume (MCV), coagulation function evaluated by the quantification of **I)** platelets (PLT) and **J)** mean platelet volume (MPV) were from mice after different treatments on day 10. I: **PBS**; II: **PPE**; III: **cL-P**. cL-P: cRGD modified liposomes loaded with PPE; cL-3: cRGD modified liposomes loaded with 3MA; asAN-P/3: autophagy inhibitor-sensitized artificially activated neutrophils. The data are expressed as mean ± SD. *$p < 0.05$, **$p < 0.01$, ***$p < 0.001$, ****$p < 0.0001$, ns: no statistical difference.
